# Revitalizing T cells: breakthroughs and challenges in overcoming T cell exhaustion

**DOI:** 10.1038/s41392-025-02327-3

**Published:** 2026-01-01

**Authors:** Yiran Wu, Yuchen Wu, Zhengyu Gao, Weixing Yu, Long Zhang, Fangfang Zhou

**Affiliations:** 1 Shangyu People’s Hospital of Shaoxing, Shaoxing University, Shaoxing, Zhejiang China; 2 Department of Oncology, the Second Affiliated Hospital of the Zhejiang University School of Medicine, Zhejiang University, Hangzhou, China; 3 MOE Laboratory of Biosystems Homeostasis and Protection and Innovation Center for Cell Signaling Network, Life Sciences Institute, Zhejiang University, Hangzhou, China; 4 School of Medicine, Hangzhou City University, Hangzhou, China; 5 The MOE Basic Research and Innovation Center for the Targeted Therapeutics of Solid Tumors, The First Affiliated Hospital, Jiangxi Medical College, Nanchang University, Nanchang, China; 6 Frontiers Medical Center, Tianfu Jincheng Laboratory, Chengdu, China; 7 The First Affiliated Hospital of Soochow University, the Institutes of Biology and Medical Sciences, Suzhou Medical College, Soochow University, Suzhou, Jiangsu China

**Keywords:** Adaptive immunity, Immunotherapy

## Abstract

T cell exhaustion is a prevalent phenomenon in chronic infections and tumor microenvironments, severely compromising the effectiveness of antitumor and antiviral immunity. In recent years, there has been significant progress in understanding the underlying mechanisms of T cell exhaustion, including external factors and intrinsic cellular changes that drive this dysfunctional state. Key external factors such as persistent antigen exposure, immune checkpoint signaling, and the cytokine milieu, as well as intrinsic changes such as altered metabolic processes, epigenetic modifications, and transcriptional reprogramming, contribute to T cell dysfunction. Emerging therapies targeting T cell exhaustion aim to restore immune function and enhance antitumor and antiviral immunity. These therapeutic strategies include immune checkpoint inhibition, cytokine therapies, metabolic reprogramming, and cell-based therapies. Despite these advancements, reversing T cell exhaustion presents several challenges, such as individual variability, resistance, and potential side effects. Furthermore, accurately assessing markers of T cell functional recovery and the long-term impacts of these therapeutic approaches remain challenging research areas. This review provides an overview of the history and milestones in T cell exhaustion research; summarizes the mechanisms of T cell exhaustion and its implications in cancer, chronic infections, and autoimmune diseases; discusses advancements and challenges in emerging therapies; and explores future research directions aimed at improving T cell function and enhancing immune responses.

## Introduction

T cells, which are essential players in the adaptive immune response, play crucial roles in recognizing and eliminating infected or malignant cells. Their ability to activate robust immune responses is integral for maintaining health and combating diseases.^1^ However, under certain conditions, particularly during chronic infections or cancer progression, T cells undergo a state of exhaustion. This phenomenon is characterized by a progressive loss of functionality, posing significant challenges in immunology and therapeutic interventions.^2–4^

Notably, T cell exhaustion is not always detrimental; it has been observed under both physiological and pathological conditions. The process underlying T cell exhaustion is complex and multifaceted and involves extrinsic factors in the immune microenvironment as well as intrinsic cellular changes. Exhausted T cells can mitigate autoimmune responses, thereby contributing to the control of autoimmune diseases.^5–7^ Furthermore, they play crucial roles in organ transplantation.^8–12^ Therefore, rather than viewing T cell exhaustion purely as a negative phenomenon, it is more appropriate to consider that T cell exhaustion can also occur under physiological conditions as a functional state that is part of normal immune regulation, tolerance, and homeostasis.^13^

Under pathological conditions such as chronic infection and cancer, prolonged antigen exposure causes sustained signaling through the T cell receptor (TCR) and associated costimulatory pathways, resulting in altered gene expression and metabolic dysfunction within T cells.^14^ Additionally, the tumor microenvironment (TME) presents unique challenges that exacerbate T cell exhaustion.^15,16^ Tumors usually create an immunosuppressive milieu characterized by the secretion of various cytokines, metabolites, and immune suppressor cells that actively inhibit T cell function. In autoimmune diseases, the situation regarding T cell exhaustion is more complex.^5,17–19^ On the one hand, T cell exhaustion may represent an adaptive mechanism by which the immune system attempts to limit excessive self-reactive immune responses. This phenomenon is considered an evolutionarily conserved response to chronic antigenic stimulation, which may be crucial for preventing immune pathology and the escalation of autoimmune conditions. However, in certain cases, exhausted T cells may fail to effectively control the autoimmune process. For example, type III hypersensitivity, also known as immune complex-mediated hypersensitivity, is an immune response characterized by the formation of antigen‒antibody complexes (immune complexes)^20^ that deposit in various tissues, leading to inflammation and tissue damage.^21–23^ This reaction involves IgG or IgM antibodies binding to soluble antigens, forming immune complexes, which then circulate in the bloodstream.^23^ When these complexes are deposited in tissues such as the kidneys, joints, and blood vessels, they trigger an inflammatory response through the activation of the complement system and the recruitment of inflammatory cells such as neutrophils. In this case, accelerated T cell exhaustion in immunocompromised individuals may lead to impaired phagocytosis of antigen–antibody immune complexes, becoming an important factor in various highly inflammatory and autoimmune diseases.^24–27^

In conclusion, T cell exhaustion is a complex and multifaceted phenomenon with significant implications for various physiological and pathological conditions. Targeting T cell exhaustion through the promotion or reversal of this state holds significant potential for immune modulation and is expected to improve patient outcomes in the context of diseases such as cancer, chronic infections, cardiovascular diseases (CVDs), neurodegenerative disorders, metabolic dysregulation, and autoimmune diseases. Future research could focus on elucidating the remaining mysteries of T cell exhaustion, especially in the context of complex disease microenvironments, and developing more precise and personalized therapies. Here, we focus on the biological basis of T cell exhaustion and the therapeutic interventions that rejuvenate exhausted T cells, restoring their effector functions.

## History and milestones in T cell exhaustion research

**Fig. 1:**
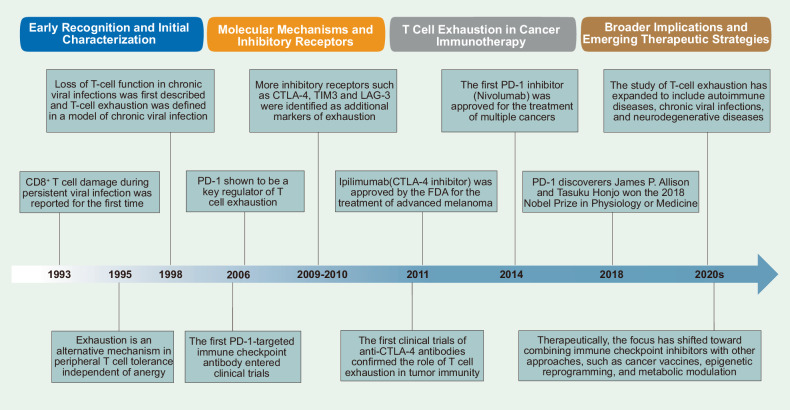
History and milestones in T cell exhaustion research. A chronological overview of key milestones in the field of T cell exhaustion research, beginning with its initial discovery and definition. This timeline highlights the progressive advancements in understanding the mechanisms underlying T cell exhaustion. Research in this area has contributed to the development of targeted immunotherapies, facilitating the formulation of more precise and effective therapeutic strategies

The initial observations of T cell dysfunction in the context of chronic infections such as lymphocytic choriomeningitis virus (LCMV) can be traced back to 1993^28,29^ (Fig. 1). In 1993, Moskophidis D et al. first described the impairment of CD8^+^ T cells during persistent viral infections.^28^ Early studies highlighted a decline in T cell effector function, but the concept of “exhaustion” has not yet been clearly defined. It is often conflated with other forms of T cell dysfunction, such as anergy.^30,31^

In 1995, Rocha et al. first proposed that anergy and exhaustion are independent mechanisms of peripheral T cell tolerance.^32^ Anergy refers to a condition where T cells, under high antigen concentrations, fail to differentiate into effector cells and instead persist as unresponsive T cells within the organism. In contrast, exhaustion occurs at lower antigen concentrations, where T cells initially differentiate into effector cells but then disappear from antigen-specific T cells.

In 1998, two research groups by Rafi Ahmed and Rolf Zinkernagel identified virus-specific CD8^+^ T cells through tetramer staining.^33,34^ They discovered that “activated” but without effector function, virus-specific CD8^+^ T cells could persist indefinitely in chronic infection in mice at remarkably high frequencies (1–2% of total CD8^+^ T cells). These T cells expressed activation/memory markers and could proliferate in vivo; however, they exhibited impaired antiviral effector functions and could not effectively control viral infection. This finding significantly advances our understanding of T cell exhaustion in the context of chronic infections.

By the mid-2000s, the mechanistic underpinnings of T cell exhaustion were better understood, especially with the discovery of key inhibitory receptors.^35–37^ The upregulation of PD-1 (Programmed Death-1) on exhausted T cells was linked to their dysfunction, and its interaction with PD-L1 (PD-1 ligand) on cancer cells or antigen-presenting cells (APCs) was shown to contribute to the suppression of T cell activity.^35,38–40^ In 2006, Barber et al. demonstrated that the blockade of PD-1 signaling in chronic LCMV infection led to the restoration of T cell function, providing compelling evidence that PD-1 is a key regulator of T cell exhaustion.^41^ In the same year, MDX1106, the first PD-1-targeted immune checkpoint antibody, entered clinical trials (NCT00441337), marking the beginning of a new era in cancer immunotherapy.^42^ During the same period, other inhibitory receptors, such as CTLA-4 (cytotoxic T lymphocyte-associated protein 4, also known as CD152), TIM3 (T cell immunoglobulin and mucin domain-containing protein 3), and LAG-3 (lymphocyte-activating gene 3), were identified as additional markers of exhaustion, further defining the molecular landscape of this phenomenon.^43^ These findings laid the groundwork for the development of immune checkpoint inhibitors (ICIs) in cancer immunotherapy, a breakthrough that would significantly influence the field in the following decade.

The application of T cell exhaustion research to cancer immunotherapy represented a major milestone in the 2010s. The success of ICIs has revolutionized the treatment of cancers such as melanoma, non-small cell lung cancer (NSCLC), and renal cell carcinoma. The first clinical trials of anti-CTL-4 antibodies, initiated in the early 2010s, confirmed the role of T cell exhaustion in antitumor immune evasion and underscored the therapeutic potential of reversing exhaustion.^44–46^ This led to the FDA (U.S. Food and Drug Administration) approval of ipilimumab (a CTLA-4 inhibitor), marking a turning point in the treatment of advanced melanoma. In 2014, the first PD-1 inhibitor (nivolumab) was approved for the treatment of multiple cancers (melanoma, NSCLC, classical Hodgkin’s lymphoma). These successes in cancer therapy prompted a broader examination of T cell exhaustion in other chronic diseases. In the 2020 s, the scope of T cell exhaustion research expanded beyond cancer and chronic viral infections, with increasing interest in the role of T cell exhaustion in autoimmune diseases, metabolic disorders, and neurodegenerative disorders.

Furthermore, recent advancements in single-cell technologies,^47^ including RNA sequencing^48,49^ and high-dimensional flow cytometry,^50^ have enabled more precise characterization of the molecular and functional changes associated with T cell exhaustion at the single-cell level. These findings provide insights into the heterogeneity of exhausted T cells and identify potential biomarkers for predicting the response to therapy.^51,52^ Therapeutically, the focus has shifted toward combining immune checkpoint inhibitors with other approaches, such as cancer vaccines, epigenetic reprogramming, and metabolic modulation. These combinatorial strategies aim not only to reverse exhaustion but also to increase the overall efficacy of immune responses.

In summary, research on T cell exhaustion has long progressed since its early recognition in chronic infections and cancer. From the identification of key inhibitory receptors and molecular mechanisms to the development of immune checkpoint inhibitors and beyond, T cell exhaustion has become a central concept in immunology. Moving forward, a deeper understanding of its regulatory networks, as well as the identification of novel therapeutic targets, will be crucial for developing more effective treatments for a wide range of diseases, from cancer to autoimmune and neurodegenerative conditions. The integration of immunological, metabolic, and epigenetic insights will likely shape the next generation of therapies aimed at overcoming T cell exhaustion and enhancing immune function.

## External factors driving T cell exhaustion

**Fig. 2:**
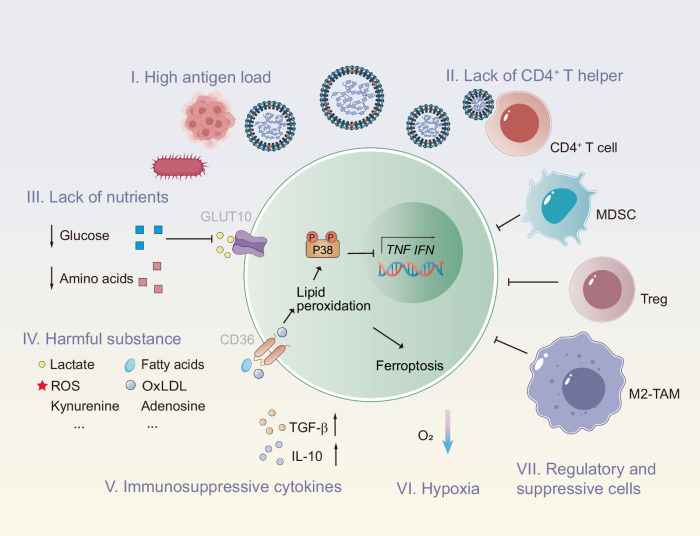
External Factors Driving T Cell Exhaustion. Chronic antigen exposure induces persistent T cell stimulation, leading to the upregulation of inhibitory receptors that limit T cell responses. Immune cytokines, such as TGF-β and IL-10, contribute to T cell suppression by establishing an immunosuppressive microenvironment. Similarly, Tregs, M2 macrophages, and MDSCs actively inhibit effector T cell function, exacerbating immune evasion. Additionally, nutrient deprivation within inflamed or tumor tissues impairs T-cell metabolism, limiting the energy resources essential for optimal immune responses. Hypoxia, which is common in tumors and chronically infected tissues, exacerbates T cell exhaustion by reducing T cell survival and proliferative capacity. The accumulation of toxic metabolites, including lactic acid and reactive oxygen species (ROS), induces cellular dysfunction and mitochondrial stress, compromising T cell responses. These overlapping and synergistic stress signals drive the exhaustion phenotype, diminishing T cell efficacy and impeding effective immune-mediated responses. These factors create a hostile environment that hampers T cell activity and compromises overall antitumor or antipathogen immunity

External environmental factors are the primary drivers of T cell exhaustion, with intrinsic cellular alterations occurring as a subsequent response to these external influences. Key contributors include prolonged antigen exposure, viral infections targeting immune cells, the immunosuppressive environment, the accumulation of harmful metabolites, nutrient deficiencies, and hypoxia. These factors collectively induce epigenetic, metabolic, and functional alterations, ultimately resulting in the exhaustion phenotype of T cells (Fig. 2).

### Persistent antigen stimulation

T cell exhaustion is a dysfunction in which T cells lose their ability to respond effectively to antigens, leading to a diminished immune response. This phenomenon is frequently observed in chronic infections; cancer; and various autoimmune, metabolic, cardiovascular, and neurodegenerative diseases. In addition to T cell exhaustion, persistent immune stimuli, including continuous exposure to pathogens, self-antigens, or inflammatory mediators, can drive T cells into other states of dysfunction, such as anergy^30,53,54^ and senescence.^55^ Each of these states presents distinct characteristics and impacts on immune function. Here, we discuss the similarities and differences between T cell exhaustion, anergy, and senescence and the role of persistent immune triggers in chronic infections, cancer, cardiovascular diseases, neurodegenerative diseases, metabolic disorders, and autoimmune diseases in driving T cell exhaustion.

#### T cell exhaustion, anergy and senescence

Anergy is another peripheral T cell tolerance mechanism that is distinct from exhaustion^30,32^ and prevents autoimmunity by inactivating potentially self-reactive T cells, which are typically induced by a lack of costimulatory signals or prolonged exposure to self-antigens in the absence of proper activation.^54^ Unlike exhaustion, anergy is often an early-stage response to inappropriate activation signals. Anergic T cells have a reduced ability to produce IL-2 and are hyporesponsive to subsequent antigenic stimulation.^30^ However, they do not typically upregulate the extensive array of inhibitory receptors expressed by exhausted T cells.^54^ Additionally, anergy can be more readily reversed than exhaustion, as it may only require the provision of proper costimulation.^31,53^ This unresponsiveness is a critical mechanism by which the immune system prevents autoimmunity under normal conditions; however, in certain autoimmune diseases, anergic T cells may become reactivated under inflammatory conditions, undermining immune tolerance.

T cell senescence refers to a state of irreversible cell cycle arrest in T cells, often driven by repeated antigenic stimulation over time.^55^ Repeated stimulation by the same antigen leads to the progressive downregulation of CD28 expression on T cells, which serves as a biological marker of immunosenescence.^55,56^ Senescent T cells have a distinct phenotype characterized by increased expression of senescence-associated-β-galactosidase (SA-β-Gal)^57,58^ and senescence-associated markers,^59^ such as CD57,^60^ CD45RA,^61^ and KLRG-1.^62^ Senescent T cells are functionally distinct from exhausted T cells in that they no longer divide or proliferate, even in response to new antigenic stimuli. These cells are functionally impaired, have a limited capacity for proliferation, and exhibit a proinflammatory phenotype, contributing to chronic inflammation and tissue damage, a phenomenon known as the senescence-associated secretory phenotype (SASP).^63,64^ Importantly, these cells accumulate over time due to both intrinsic cellular mechanisms (e.g., telomere shortening and DNA damage) and extrinsic factors (e.g., chronic inflammation), particularly in older individuals. Senescent T cells are implicated in several age-related diseases, including cardiovascular disease, neurodegenerative conditions, and metabolic disorders. In contrast to exhausted T cells, which can be reinvigorated by checkpoint inhibition, senescent T cells are more difficult to reverse. Strategies targeting the SASP or using senolytic therapies^65^ may offer potential avenues for therapeutic intervention.

#### Chronic infections and cancer

Studies using the LCMV mouse model have demonstrated that antiviral therapy initiated early in infection preserves T cell functionality, whereas prolonged antigen exposure prevents T cells from recovering their normal memory and stem-like properties once antigenic stimuli are removed.^66,67^ These results underscore the critical influence of the intensity and duration of antigen exposure on T cell functionality, with persistent antigen exposure directly correlating with the development of T cell dysfunction. As viral antigens persist in the body, they continue to drive T cell activation. Over time, this persistent stimulation results in the upregulation of inhibitory receptors, leading to the characteristic exhausted T cell phenotype.^3^ These receptors, such as PD-1, CTLA-4, and TIM-3, limit T cell activity, preventing excessive immune responses that can damage host tissues.^68^ However, regarding chronic infections and cancers, the regulation of T cell activity by these inhibitory receptors contributes to a state of functional impairment, wherein T cells exhibit diminished proliferation, reduced cytokine production, and an overall decline in effector functions. Hence, exhausted T cells become less effective at controlling pathogen replication or inhibiting cancer growth, allowing these threats to persist and evade immune surveillance.

#### Cardiovascular diseases

In cardiovascular diseases, persistent immune activation often arises from chronic inflammation,^69,70^ a hallmark of atherosclerosis, myocardial infarction, and heart failure.^71^ Numerous preclinical and clinical studies have demonstrated that T cell-mediated immunity plays a central role in the pathogenesis of CVD.^72,73^ Endothelial dysfunction and the accumulation of oxidized low-density lipoproteins (oxLDLs) promote a continuous immune response.^74,75^ In the context of atherosclerosis, T cells, particularly CD4^+^ T helper cells, are constantly exposed to self-antigens presented by the activated endothelium^76–78^ and foam cells.^72,79,80^ Under sustained antigen stimulation, T cells may enter a state of exhaustion to mitigate self-damage caused by excessive immune activation. Gene expression data from CITE-seq and single-cell RNA sequencing revealed that human atherosclerotic plaques are enriched with T cells exhibiting cytotoxicity, activation, and exhaustion markers.^81,82^ Furthermore, the adoptive transfer of cytotoxic and proinflammatory T lymphocytes accelerates the formation of atherosclerotic plaques,^83,84^ whereas the transfer of regulatory T (Treg) cells exerts a protective effect in murine models.^85–87^ Notably, T cells in human atherosclerotic plaques exhibit increased PD-1 expression.^81^ Disruption of the PD-1 pathway enhances atherosclerotic lesion progression and inflammation,^88^ whereas activation of the PD-1/PD-L1 pathway inhibits atherosclerotic lesion development in murine models.^89^

However, the specific role of T cell exhaustion in cardiovascular lesions remains incompletely understood. As targeting regulatory T cells has emerged as a promising therapeutic strategy,^90–93^ advancing research into T cell exhaustion in CVD may offer novel therapeutic avenues for modulating immune-mediated inflammation in atherosclerosis.

#### Neurodegenerative diseases

Neurodegenerative diseases, such as Alzheimer’s disease and Parkinson’s disease, are characterized by chronic neuroinflammation^94,95^ and the accumulation of misfolded proteins (e.g., amyloid-β, α-synuclein).^96,97^ An increasing body of evidence highlights the involvement of T cells in central nervous system (CNS)-specific inflammatory responses associated with neurodegenerative processes.^98–101^ Antigen-specific T cells have been identified in the blood and cerebrospinal fluid (CSF) of patients with neurodegenerative diseases.^99,100,102,103^ Although the entry of immune cells into the CNS is tightly restricted by barriers such as the blood–brain barrier, blood–CSF, and CSF–brain barrier,^104–106^ antigens may be exported from the CNS and presented in an immunogenic manner in draining lymph nodes.^107–109^ These antigens can originate from virus-infected CNS cells or self-antigens released due to intrinsic oligodendrocyte disorders or degenerative processes within the CNS.^107^ In animal models of Alzheimer’s disease, it has been proposed that CNS antigen-specific T cells exert a protective effect on the choroid plexus^110,111^ and that breaking immune tolerance can mitigate Alzheimer’s disease pathology.^111^ Nevertheless, prolonged exposure to amyloid plaques can trigger sustained activation of T cells, fostering a chronic neuroinflammatory environment. With chronic exposure to these antigens in the context of the CNS microenvironment, which has unique immune-regulatory properties, T cells may undergo a transition to an exhausted phenotype. The role of exhausted T cells in neurodegenerative diseases is still not fully understood, but exhausted T cells may be unable to effectively eliminate self-antigens released during neurodegenerative processes, thereby accelerating neurodegeneration and functional decline. Therapeutic strategies that target the adaptive immune system, such as vaccination and monoclonal antibodies, have strong potential for alleviating amyloid plaque build-up and improving cognition in Alzheimer’s disease patients.^112–115^

#### Metabolic disorders

In metabolic disorders such as obesity, type 2 diabetes, and nonalcoholic fatty liver disease (NAFLD), chronic low-grade inflammation plays a central role in disease pathogenesis.^116–120^ Adipose tissue, particularly in obese individuals, is an active endocrine organ that secretes proinflammatory cytokines such as IFN-γ (interferon-γ), TNF-α (tumor necrosis factor-α), and IL-6 (interleukin-6).^121–123^ These factors contribute to the recruitment and persistent activation of T cells. In the case of type 2 diabetes, for example, elevated levels of free fatty acids and hyperglycemia create an environment of chronic immune activation.^124–126^ CD8^+^ T cells play a critical role in initiating the inflammatory cascade within obese adipose tissue. The infiltration of CD8^+^ T cells precedes macrophage accumulation in adipose tissue, and upon activation, these cells initiate and propagate the inflammatory response. This includes the recruitment of monocytes and macrophages to the site of inflammation within obese adipose tissue.^123^ As T cells are continually exposed to these inflammatory mediators, they undergo a gradual process of exhaustion, as evidenced by the upregulation of inhibitory receptors (e.g., PD-1) and the reduction in their ability to produce proinflammatory cytokines such as IFN-γ and IL-2.^123,127,128^

T cell exhaustion in obesity may function as a physiological feedback mechanism to mitigate the metabolic inflammation sustained by macrophages. Studies have shown that CD8^+^ T cell depletion can improve systemic insulin resistance, whereas the adoptive transfer of CD8^+^ T cells to CD8-deficient mice exacerbates adipose inflammation.^123^ These findings strongly suggest that CD8^+^ T cell-dependent adipose inflammation influences systemic metabolism. However, exhausted T cells are less effective at reversing chronic low-grade inflammation in adipose tissue, thus allowing for the persistence of the inflammatory response. Therefore, further research is needed to fully elucidate the impact of T cell exhaustion on obesity-induced chronic low-grade inflammation.

#### Autoimmune diseases

Autoimmune diseases, such as type 1 diabetes (T1D), systemic lupus erythematosus (SLE), rheumatoid arthritis, and multiple sclerosis, are characterized by the aberrant activation of the immune system against self-antigens.^129–132^ Many autoimmune diseases are driven by self-reactive T cells, which evade central and peripheral tolerance and actively target their own tissues. In these cases, self-reactive T cells are chronically stimulated by self-antigens and may develop an exhaustion phenotype with limited function.^4,6,19^ This can serve as an additional regulatory mechanism to help limit excessive T cell-mediated damage. Exhaustion-like inactivation of T cells is a common pathway for self-antigen-specific T cells in chronic autoimmune diseases and has been detected in autoantigen-specific CD8^+^ T cells and helper T cells.^17,19,133,134^ Several studies have shown that higher levels of T cell exhaustion are associated with a better prognosis in patients with autoimmune diseases.^5,6,12^ Building on these findings, the concept of therapeutic exhaustion has been proposed, which involves promoting T cell exhaustion within the organism to restore immune tolerance and regulate immune balance, thereby suppressing the development of autoimmune diseases.^6,135,136^ Currently, research on therapeutic exhaustion remains limited, although several studies have reported the induction of autoimmune remission and tolerance in type 1 diabetes patients through the promotion of T cell exhaustion.^137–142^ For example, teplizumab, a monoclonal anti-CD3 antibody, has been shown in clinical studies to induce features of T cell exhaustion in CD8 effector memory T cells, leading to a median delay of 24 months in the overall development of type 1 diabetes.^138^ However, the potential adverse effects of such approaches have yet to be fully elucidated. To effectively translate therapeutic exhaustion into immunotherapy for autoimmune diseases, a deeper understanding of the molecular mechanisms underlying T cell exhaustion is essential, and extensive preclinical and clinical studies are needed to evaluate its therapeutic potential.

In summary, chronic exposure to self-antigens, inflammatory mediators, and immune checkpoints creates a pathological environment that drives T cell dysfunction. This state of exhaustion can both alleviate autoimmune responses and inhibit disease progression, but it may also impair the immune system’s ability to resolve inflammation, eliminate pathological factors, or maintain tissue homeostasis, potentially leading to disease progression and worsened clinical outcomes. Further research is needed to explore the molecular mechanisms affecting T cell exhaustion and assess the physiological importance of T-cell exhaustion in immune regulation and disease progression.

### Direct attack on T cells

Chronic infections by viruses, such as HIV (human immunodeficiency virus), comprehensively reveal how viruses impair T cell activity and promote exhaustion. HIV specifically targets CD4^+^ T helper cells, which play a vital role in orchestrating immune responses.^143^ The loss of CD4^+^ T cells compromises CD8^+^ cytotoxic T cell responses and diminishes CD8^+^ T cell-mediated control of chronic viral infections.^144–146^

During viral replication, HIV-infected cells express viral proteins that alter the behavior of nearby uninfected T cells. One of the key viral proteins involved in this process is Nef (negative factor), which plays a critical role in the modulation of host immune signaling. The HIV protein Nef downregulates critical surface molecules on infected cells, such as CD4 and major histocompatibility complex class I (MHCI), impairing the presentation of HIV-derived peptides to cytotoxic T cells.^147–149^ This disruption leads to the suboptimal activation of CD8^+^ T cells, promoting their exhaustion through persistent, ineffective signaling. Nef mediates the downregulation of CD4 at the cell surface through a series of intricate molecular processes. Initially, Nef directly binds to the cytoplasmic tail of CD4,^150,151^ specifically interacting with a highly conserved sequence within the intracellular domain of CD4 located between its transmembrane and cytoplasmic regions.^152,153^ Upon binding, Nef recruits the host cell machinery to facilitate the internalization of CD4 via the clathrin-mediated endocytosis pathway.^154,155^ This process involves the formation of clathrin-coated vesicles that encapsulate CD4 molecules from the plasma membrane. Following endocytosis, Nef directs the trafficking of internalized CD4 molecules to late endosomes and lysosomes,^156^ where they are subsequently degraded. This degradation process is mediated through interactions between Nef and host cell adaptor proteins, including the adaptor protein 2 (AP-2) complex,^157,158^ which plays a pivotal role in clathrin-mediated endocytosis, as well as Rab GTPases that regulate vesicular trafficking.

In addition to its effects on CD4, Nef also interacts with the cytoplasmic tail of MHC-I molecules,^156,159^ specifically the HLA-A and HLA-B alleles.^160^ Following Nef binding, MHC-I molecules are retained within the trans-Golgi network (TGN) and fail to reach the plasma membrane,^161,162^ thereby preventing their surface expression and hindering the presentation of viral antigens to cytotoxic T lymphocytes (CTLs). Furthermore, Nef interacts with various host cell proteins to promote the downregulation of MHCI. For example, Nef recruits AP-1 to facilitate the endocytosis and subsequent degradation of MHCI molecules.^163,164^

Human herpesvirus-6 (HHV-6), a type of lymphotropic virus, preferentially infects CD4^+^ T cells, causing significant functional alterations in infected T cells and increasing the risk of lymphoproliferative disorders.^165–167^ HHV-6 infection can induce G(2)/M cell cycle arrest in infected T cells through multiple molecular regulatory mechanisms, thereby inhibiting lymphocyte proliferation.^166,168–170^ Mechanistically, HHV-6 infection leads to the upregulation of Wee1 expression and the inactivation of Cdc25C, which results in inhibitory phosphorylation at Tyr15, subsequently causing a significant reduction in the activity of the Cdc2‒cyclin B1 complex.^166^ Furthermore, the decreased activity of this complex is partly driven by p53-dependent upregulation of the cell cycle regulator p21. HHV-6A infection also activates the DNA damage checkpoint kinases Chk2 and Chk1,^166^ further contributing to cell cycle perturbations.

Similarly, tumors exploit multiple strategies to directly target T cells and promote their exhaustion. Recent studies suggest that tumor cells induce direct cytotoxic effects on T cells by releasing exosomal proteins and RNA.^171,172^ For example, exosomes released by head and neck cancer-derived cell lines induce CD8^+^ T cell suppression, further impairing the antitumor immune response.^173^ Mass spectrometry analysis revealed that exosomes with immunosuppressive activity are enriched with the immune regulatory protein galectin-1. In contrast, exosomes derived from Galectin-1 gene knockout tumor cells were unable to induce T cell suppression. Additionally, RNA isolated from exosomes with T cell inhibitory properties was found to partially inhibit T cell function upon transfection into CD8^+^ T cells. Collectively, these results suggest that the immunosuppressive effects of tumor-derived exosomes may result from synergistic interactions between exosomal proteins and RNA. However, the precise molecular mechanisms driving this phenomenon remain to be elucidated. These mechanisms highlight how viral infections and tumors directly manipulate T cell function, causing the onset of T cell exhaustion and the consequent failure of immune responses.

### Unfavorable microenvironment

#### Immunosuppressive environment

The unfavorable microenvironment is another significant contributor to T cell exhaustion and includes high levels of immunosuppressive cytokines, harmful metabolites, nutrient deficiency, and low oxygen levels, creating an immunosuppressive environment. Chronic infections, including those caused by HIV, HCV, and certain bacterial pathogens, are usually characterized by persistent inflammation and a unique cytokine microenvironment. Inflammation, a complex biological response to harmful stimuli, involves the activation of various immune cells, which induces cytokine release. The cytokine environment activates T cells in the initial phases of the immune response; however, long-term exposure to certain cytokines, particularly those that are immunosuppressive, causes T cell dysfunction or exhaustion. Among the crucial cytokines involved in this process, IL-10 plays a key role in immune regulation. Induced through the STAT (Signal Transducer and Activator of Transcription) family of transcription factors, IL-10 suppresses T cell activation and is commonly elevated at the expression level during chronic infections and cancers. Various immune cells, including monocytes, B cells, dendritic cells (DCs), nonregulatory CD4^+^ T cells, and CD8^+^ T cells, produce IL-10 in response to persistent antigenic stimulation.^174,175^ Notably, inhibiting IL-10 signaling in the context of chronic viral infections reportedly prevents or even reverses T cell dysfunction, resulting in improved viral control and improved immune responses.^176,177^ Another key cytokine involved in T cell exhaustion is TGF-β (Transforming Growth Factor-beta). This cytokine is crucial for suppressing immune responses and promoting T cell exhaustion.^178,179^

TGF-β is a cytokine family composed of three isoforms, namely, TGF-β1, TGF-β2, and TGF-β3, which play pivotal roles in immune regulation.^180,181^ TGF-β signals through its receptors, primarily type I (TGF-βR1) and type II (TGF-βR2) serine/threonine kinase receptors. Upon binding to TGF-βR2, it recruits and phosphorylates TGF-βR1, which then activates the intracellular signaling cascade through the SMAD (Sma- and Mad-related protein) family of proteins.^182–184^ SMAD2 and SMAD3 are phosphorylated by activated TGF-βR1, and they subsequently form a complex with SMAD4.^185^ This SMAD complex translocates to the nucleus where it regulates the transcription of target genes involved in immune suppression, cellular differentiation, and tissue fibrosis. In addition to SMAD-dependent pathways, TGF-β signaling also interacts with non-SMAD pathways, such as MAPK (mitogen-activated protein kinase) and PI3K/AKT (phosphoinositide 3-kinase/protein kinase B),^186–189^ further modulating immune cell function and contributing to the persistence of T cell dysfunction.

In the context of T cell exhaustion, TGF-β signaling plays a central role.^179,190,191^ TGF-β impedes the production of key cytokines, such as IL-2 and IFN-γ, which are essential for T cell proliferation and effector function.^192,193^ This suppression of effector T cell responses may be partly mediated by TGF-β-induced transcriptional changes, leading to changes in the expression of key transcription factors such as T-bet and Eomes,^194,195^ which are essential for maintaining T cell function. Furthermore, TGF-β promotes the differentiation of naïve T cells into regulatory T cells,^196^ increasing the immunosuppressive network and fostering T cell exhaustion. Targeted inhibition of TGF-β signaling in T cells leads to an increase in the population of antigen-specific CD8^+^ T cells, enhancing viral control and improving immune function.^191,197,198^

The TME involves dynamic interactions between tumor cells, immune cells, and various soluble factors, creating an immunosuppressive environment. Cytokines within the TME influence the recruitment of mononuclear cells from the bloodstream to the tumor site, where they are transformed into tumor-associated macrophages (TAMs) through the action of chemokines and cytokines.^199,200^ TAMs are abundant in the TME and are important in shaping T cell responses. They adopt a proinflammatory (M1) or anti-inflammatory (M2) phenotype.^201^ M1 TAMs release proinflammatory cytokines such as IL-12, TNF-α, and IFN-γ, which stimulate immune responses. In contrast, M2 TAMs secrete immunosuppressive cytokines (e.g., IL-10 and TGF-β) and express immune checkpoint ligands (e.g., PD-L1) that inhibit T cell activity and promote exhaustion. As cancer progresses, M1 macrophages gradually shift toward the M2 type, and an increased presence of M2-type TAMs is associated with a poor prognosis. Additionally, myeloid-derived suppressor cells (MDSCs) constitute a key population within the TME that suppresses T cell function. MDSCs promote Treg expansion and enhance Treg-mediated immune suppression.^202^ MDSCs also produce reactive oxygen species (ROS), deplete cysteine in the microenvironment, and increase the activity of inducible nitric oxide synthase (iNOS) and arginase-1 to consume L-arginine, inhibiting T cell generation.^203,204^

#### Accumulation of harmful metabolites

Abnormal metabolism is a hallmark of cancer that promotes cancer cell growth and survival and fosters an immunosuppressive microenvironment. Many tumors undergo metabolic reprogramming, referred to as the Warburg effect, wherein they predominantly depend on glycolysis for energy, even under aerobic conditions.^205^ This metabolic switch causes the accumulation of lactate, acidifying the TME. Acidic conditions directly inhibit TCR signaling pathways, reduce cytokine production and cytotoxicity,^206–208^ and alter the migratory capacities of T cells, making it more challenging for them to reach and infiltrate tumor sites.^209^ Furthermore, increased lactate concentrations in the TME inhibit the function of GLUT1, a crucial glucose transporter for CD8^+^ T cell activation and antitumor immune responses. This disruption impedes glucose uptake by CD8^+^ T cells, thereby impairing their antitumor activity.^210^ Studies in various cancers have shown that high lactate levels are correlated with poor prognosis and diminished T cell responses.^211,212^ Targeting lactate production with inhibitors reportedly enhances T cell functionality and improves antitumor immunity in preclinical models.^213–215^

Moreover, tumor cells frequently exhibit altered lipid metabolism, leading to lipid accumulation within the TME, which significantly impacts T cell function and survival.^216^ Therefore, targeting lipid metabolism pathways has surfaced as a promising therapeutic strategy to enhance T cell responses.^217^ Altered lipid metabolism in tumors induces the production of immunosuppressive lipids, such as prostaglandins, cholesterol, oxidized fatty acids, and other bioactive lipids, which negatively impact T cell function and induce suppressive functions in MDSCs.^218–221^ For example, the accumulation of very long-chain fatty acids or cholesterol contributes to the impaired function of tumor-infiltrating CD8^+^ T cells.^220,222^ Recent studies by German and Swiss researchers have demonstrated that tumor-derived prostaglandin E2 (PEG_2_) inhibits the CD8^+^ T cells by disrupting IL-2 signaling, hindering the expansion of stem cell-like CD8^+^ T cells, reducing the number of CD8^+^ T cells infiltrating tumors, and resulting in cell death owing to mitochondrial dysfunction.^218,219^ Furthermore, increased lipid uptake by intertumoral Treg cells enhances their persistence and suppressive functions.^223^

Additionally, excessive ROS production is a widespread metabolic change observed in the TME. Increased ROS levels induce oxidative stress, impairing T cell signaling pathways crucial for activation and function and contributing to exhaustion.^224,225^ Furthermore, dysregulated lipid metabolism, coupled with increased ROS production, induces lipid peroxidation, generating a significant quantity of “harmful lipids.” These oxidized lipids are subsequently taken up by CD8^+^ T cells through CD36 receptors on the cell surface, triggering further lipid peroxidation and activating downstream signaling pathways, such as p38 kinase. This cascade negatively impacts the antitumor activity of immune cells.^226^

#### Nutrient deficiency

Tumors alter their metabolism and create a competitive environment for nutrients and resources among immune cells, causing immune suppression and T cell exhaustion. For example, cancer cells usually consume abundant glucose, leaving a small amount available for infiltrating T cells.^227,228^ This depletion of glucose hinders T cell activation and proliferation, initiating their exhaustion. The availability of amino acids is another critical factor for T cell activation and function. Tumors frequently sequester vital amino acids, particularly glutamine and tryptophan, leading to competition between cancer cells and T cells, which affects T cell metabolism.^229,230^ T cell activation and proliferation rely on glutamine as a critical nutrient. It is a nitrogen source for nucleotide synthesis and supports the tricarboxylic acid (TCA) cycle for energy production.^231^ In glutamine-depleted environments, T cells exhibit reduced proliferation and impaired effector functions.^231^ Indoleamine 2,3-dioxygenase (IDO) is an enzyme expressed in some tumors and macrophages that depletes tryptophan, a crucial amino acid required for T cell function.^232,233^ Tryptophan catabolism by the enzyme IDO causes kynurenine accumulation, which has immunosuppressive effects and prevents the activation of CD8^+^ and CD4^+^ effector T cells, inhibits natural killer (NK) cell function, and stimulates Treg activation.^234,235^ IDO inhibition in cancer cells indicates a significantly better prognosis^233^ and has been shown to rejuvenate T cell function and boost antitumor immunity in preclinical models.^236–238^

#### Hypoxia

Finally, the TME is a hypoxic environment resulting from inadequate blood supply.^239^ Tumors adapt by activating hypoxia-inducible factors (HIFs), which drive metabolic reprogramming to support their growth and survival. HIF1α promotes the expression of immune checkpoint ligands such as PD-L1 on tumor cells, contributing to T cell exhaustion.^240^ Moreover, hypoxia causes the accumulation of adenosine, a metabolite that suppresses T cell activity.^241^ Additionally, metabolic pressure originating from mitochondria under hypoxic conditions accelerates terminal cell differentiation and increases ROS levels in T cells, leading to severe T cell dysfunction and failure.^242^

## Intrinsic cellular changes underlying T cell exhaustion

**Fig. 3:**
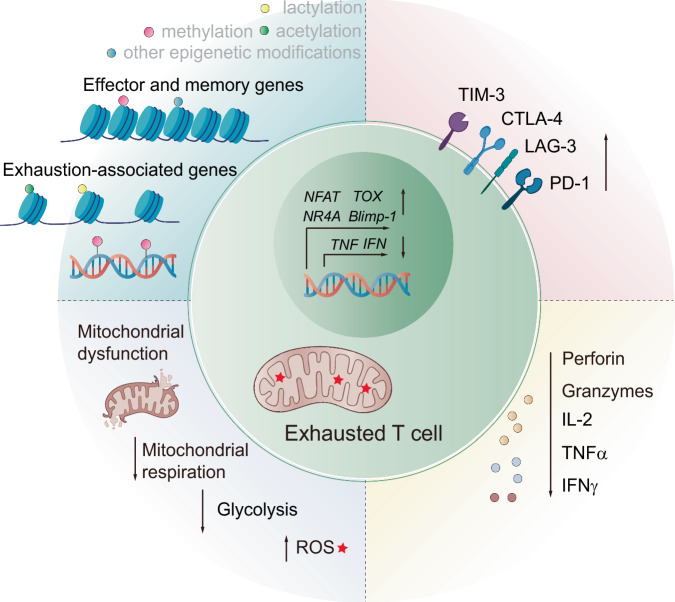
Intrinsic Cellular Changes Underlying T Cell Exhaustion. T cell exhaustion is characterized by the upregulation of inhibitory receptors, such as PD-1, CTLA-4, and TIM-3, which serve as defining hallmarks of this phenotype. These inhibitory receptors play crucial roles in the modulation of T cell responses by limiting activation and promoting immune evasion. In addition to altered receptor expression, the cytokine profile of exhausted T cells is characterized by a diminished capacity to produce proinflammatory cytokines, including IL-2, TNF-α, and IFN-γ, whereas the expression of immunosuppressive cytokines, such as IL-10 and TGF-β, further contributes to immune suppression. Exhausted T cells also exhibit distinct metabolic changes, including impaired glycolysis and mitochondrial dysfunction, which lead to energy deficits and hinder T cell proliferation and effector function. At the molecular level, the transcriptional profile of exhausted T cells revealed the upregulation of genes associated with cell death and immune tolerance and the downregulation of those responsible for effective immune responses. Moreover, epigenetic modifications, such as altered histone modifications and DNA methylation patterns, play a key role in the stable maintenance of the exhaustion state, rendering the phenotype resistant to reversal

T cell exhaustion is characterized by several significant features distinguishing exhausted T cells from their functional counterparts.^2–4^ In this section, we explore the biological characteristics of exhausted T cells, including changes in surface markers, cytokines, metabolism, transcriptional profiles, and epigenetic modifications that define this state (Fig. 3).

### Upregulation of inhibitory receptors

A key feature of T cell exhaustion is the upregulation of inhibitory receptors, which are essential for maintaining immune homeostasis and preventing excessive inflammation. However, when overexpressed under persistent antigen exposure, these receptors impair T cell function.^68^ The cumulative presence of these inhibitory receptors establishes a “checkpoint” mechanism that acts as a negative modulator of T cell activation and is increasingly recognized as a promising therapeutic target for treating solid tumors and leukemia. Some crucial surface markers associated with exhausted T cells are discussed here.

PD-1 is among the most well-studied inhibitory receptors.^38,243–245^ Its expression increases on T cells during chronic viral infections and cancers. When engaged by its ligands (PD-L1 and PD-L2), PD-1 transmits inhibitory signals through the recruitment of phosphatases such as SHP-2 (Src Homology 2 Domain-Containing Phosphatase 2), which reduce T cell proliferation and cytokine production. Similarly, CTLA-4 is an inhibitory receptor that competes with CD28 for binding to B7 molecules (CD80/CD86) on antigen-presenting cells. While CD28 engagement provides costimulatory signals essential for T cell activation, CTLA-4 inhibits this process by downregulating costimulatory signals, promoting T cell anergy or exhaustion. The expression of CTLA-4 early in immune responses can prevent overactivation, whereas its upregulation in chronic infection or cancers contributes to T cell dysfunction.^36,246,247^

TIM-3, also known as CD366 or HAVCR2, is expressed on exhausted CD4^+^ and CD8^+^ T cells and has multiple ligands, such as galectin-9 (primary ligand), CEACAM-1, HMGB1, and phosphatidylserine. TIM-3 engagement by these ligands leads to reduced T cell effector functions and fosters an immunosuppressive environment.^248,249^

LAG-3, which is structurally similar to CD4, binds to MHC class II molecules with high affinity,^250^ and its engagement triggers inhibitory signals that dampen T cell activation.^251,252^ LAG-3 upregulation is correlated with sustained antigen presentation and chronic stimulation.^43^ LAG-3 disrupts the interaction between the tyrosine kinase Lck and the coreceptors CD4 or CD8 through a conserved cytoplasmic tail, thereby limiting coreceptor–TCR signaling and T cell activation.^253,254^

TIGIT (T cell immunoglobulin and the ITIM domain), which is expressed primarily in T cells,^255^ NK cells,^256^ and regulatory T cells,^257^ binds to the poliovirus receptor (PVR/CD155) and inhibits T cell activation. Upon binding to PVR, phosphorylated TIGIT recruits Grb2 and β-arrestin, which subsequently recruit SHIP1 and SHP2 to downregulate critical signaling pathways (e.g., PI3K/NF-κB).^256,258,259^

The induction and regulation of immune checkpoints are subject to temporal and spatial control by the local immune microenvironment. A thorough understanding of these regulatory mechanisms is essential for the development of therapeutic strategies aimed at modulating immune checkpoint expression to enhance immune responses. Specifically, from a temporal perspective, immune checkpoints play pivotal roles early in the immune response by balancing T cell activation and preventing autoimmunity, whereas in chronic conditions, they contribute to T cell exhaustion and immune evasion. From a spatial perspective, the expression of immune checkpoints is dynamically regulated across various tissues and is responsive to environmental stimuli, including sustained antigen exposure, inflammation, and cytokine signaling.

In peripheral tissues under normal physiological conditions, immune checkpoint expression is typically low in naïve and resting T cells.^260,261^ These tissues serve as sites for T cell surveillance, where immune checkpoints are often maintained at baseline levels to prevent unnecessary immune activation. However, even under steady-state conditions, certain tissues, such as lymphoid organs, may exhibit increased expression of immune checkpoint molecules, especially regulatory T cells^257,262^ and innate lymphoid cells,^263,264^ which modulate immune tolerance.

Upon encountering their cognate antigen presented by antigen-presenting cells, T cells undergo activation and proliferation.^260^ Early immune checkpoint molecules such as CTLA-4 are upregulated following T cell activation to maintain immune homeostasis and prevent autoimmunity by limiting the duration and intensity of the immune response.^265–267^ At this stage, costimulatory signals (e.g., CD28-CD80/86) and inhibitory signals (e.g., CTLA-4) are balanced to regulate T cell activation and prevent excessive immune responses. Furthermore, upon antigen recognition, T cells migrate to the site of infection, cancer, or injury, where they encounter a range of inflammatory cytokines and signals that modulate their function. In the local immune microenvironment, the expression of immune checkpoints such as CTLA-4 and PD-1 is induced in activated T cells, dampening T cell effector functions and preventing excessive tissue damage.

After the resolution of infection or the removal of tumor cells, the immune system needs to return to a homeostatic state. During this phase, the expression of immune checkpoints may return to baseline levels, and T cells are restored to a functional, quiescent state. However, in cases of chronic disease or cancer, persistent antigen exposure leads to sustained T cell activation, often resulting in a state known as T cell exhaustion. During this phase, the expression of multiple immune checkpoint molecules, including PD-1, LAG-3, and TIGIT, becomes significantly upregulated.^268,269^ T cells in this exhausted state exhibit reduced proliferative capacity, diminished cytokine production, and impaired cytotoxic activity, thus contributing to immune evasion.

### Changes in effector cytokine production

Exhausted T cells exhibit a reduced ability to produce effector cytokines, which are critical for efficient immune responses, compromising their ability to combat persistent pathogens. For example, during infections by HIV or HBV, the failure of T cells to sustain their effector functions causes viral persistence and ongoing tissue damage,^270,271^ exacerbating the pathological state. This inability to combat infection causes a vicious cycle of chronic inflammation and progressive T cell exhaustion, compromising the overall strength of immune responses. In chronically infected mice, CD8^+^ T cell dysfunction occurs in a hierarchical pattern.^272^ Impaired IL-2 production is the first function to be compromised in exhausted T cells, weakening immune responses. This is followed by a reduced ability to produce TNF-α, a crucial cytokine that kills or inhibits cancer cells. Finally, the loss of IFN-γ production represents the last dysfunctional phenotype, as this cytokine is crucial for enhancing the immune response against pathogens. Furthermore, exhausted CD8^+^ T cells exhibit decreased levels of perforin and granzymes, which are essential for the effective elimination of infected or malignant cells.^2^ Notably, even at most terminal stages, exhausted CD8^+^ T cells retain some cytotoxic activity and secrete granzyme B, thereby maintaining a level of immune surveillance.^273^

### Metabolic reprogramming in exhausted T cells

Metabolism plays a crucial role in determining T cell fate and function, with T cell exhaustion being associated with metabolic changes that profoundly impair T cell functional capacity.^274^ Exhausted T cells exhibit impaired mitochondrial respiration and glycolysis,^275,276^ and this metabolic reprogramming critically influences energy availability and T cell functionality. In contrast, during normal activation, T cells upregulate glycolysis to satisfy the increased energy demands required for rapid proliferation and effector functions.^277,278^ While oxidative phosphorylation is more efficient in terms of ATP yield, the shift toward glycolysis in CD8^+^ T cells during acute infection reflects an adaptive response to the high-energy requirements of immune activation. This metabolic reprogramming enables T cells to rapidly generate energy, synthesize essential biomolecules, and activate an effective immune response.^279^ This metabolic flexibility enables T cells to function more effectively in different immune environments. However, in exhausted T cells, this glycolytic pathway is impaired, resulting in insufficient production of energy to sustain their activity. This metabolic dysfunction causes reduced ATP levels and diminished effector functions, contributing to the loss of T cell efficacy. Moreover, exhausted T cells display altered activity of crucial metabolic sensors such as mammalian target of rapamycin (mTOR) and AMP-activated protein kinase (AMPK).^280,281^ These sensors are crucial in regulating cellular metabolism and growth. The dysregulation of these metabolic pathways further perpetuates the exhaustion state, compounding the challenges in restoring T cell functionality in chronic infections and malignancies.

mTOR activity plays a crucial role in maintaining the balance and functional adaptability of T cells.^282–285^ During chronic LCMV infection in mice, the activation of AKT and mTOR in antiviral cytotoxic T lymphocytes is impaired, leading to the upregulation of the transcription factor FoxO1.^286^ FoxO1, which acts as a transcriptional activator of PD-1,^287,288^ promotes the differentiation of terminally exhausted T cells. Furthermore, the competitive consumption of amino acids and glucose by tumor cells results in a state of nutrient deficiency in the TME, which subsequently inhibits mTOR activity.^141,227^ This suppression of mTOR signaling favors the regulatory function of Tregs while attenuating effector T cell activation.

On the other hand, nutrient deficiency experienced by T cells can lead to AMPK activation.^289,290^ AMPK activation acts as a cellular energy sensor, promoting catabolic pathways such as fatty acid oxidation and autophagy to restore the cellular energy balance.^291–293^ However, prolonged or sustained activation of AMPK in exhausted T cells may also suppress anabolic processes in part through its inhibition of the mTORC1 pathway,^294–296^ including protein synthesis and cell growth, further contributing to the dysfunction and impaired effector capabilities of these cells.

### Transcriptional and epigenetic regulation

The transcriptional and epigenetic profiles of exhausted T cells differ markedly from those of their active counterparts.^297^ Transcription factors such as NFAT (nuclear factor of activated T cells), TOX (thymocyte selection-associated high mobility group box), and NARF (nuclear receptor transcription factors), among others, which regulate T cell differentiation and function, exhibit distinct expression patterns in exhausted T cells. TCR-induced signals activate the NFAT family of transcription factors, which interact with other regulatory factors to modulate T cell activation and effector differentiation.^298–300^ Notably, CD8^+^ T cells lacking NFAT cannot express depletion-associated inhibitory receptors.^300^ In addition, TOX^301–303^ and NR4A^304,305^ family members, secondary transcription factors induced by NFAT, have surfaced as pivotal regulators of T cell exhaustion. The expression of these factors is upregulated in exhausted T cells and contributes to the maintenance of the dysfunctional state by promoting the expression of inhibitory receptors. Similarly, Blimp-1 (B lymphocyte-induced maturation protein 1) is associated with the terminal differentiation of effector T cells and may exhibit dysregulated expression in the exhausted state, exacerbating T cell dysfunction.^306–308^ Moreover, the subcellular localization of transcription factors, such as T-bet and Eomesodermin (Eomes), is crucial for their regulatory activity, particularly in exhausted T cells.^309^ These transcriptional changes alter the functional capabilities of T cells. Thus, exhausted T cells demonstrate a reduced ability to respond effectively to antigens, contributing to their dysfunction in immune responses.

Additionally, there are significant differences at the epigenetic level between exhausted and activated CD8^+^ T cells. For example, the chromatin regions involved in regulating gene expression associated with the exhausted state are in a more open state.^310,311^ This open chromatin structure promotes interactions between cis-acting elements and trans-acting factors, improving the efficiency of specific gene transcription activities. This phenomenon, referred to as chromatin accessibility, designates these highly accessible chromatin regions as open chromatin regions (OCRs). In contrast, exhausted CD8^+^ T cells present highly condensed chromatin structures in the regions involved in effector function and memory formation, impeding the transcription of critical genes for T cell effector functions.^105^ Consequently, manipulating chromatin remodeling pathways may offer promising strategies for reversing T cell exhaustion and restoring T cell efficacy. Notably, chromatin remodeling complexes, such as the SWI/SNF complex, are vital for modifying chromatin architecture and influencing gene accessibility, significantly shaping the epigenetic landscape of exhausted T cells.^312,313^

Altered gene expression is usually accompanied by epigenetic modifications that reinforce the exhausted state, making the reversal of this state challenging. Epigenetic modifications play crucial roles in the progression of T cell exhaustion.^297^ These modifications involve DNA or histone modifications, processes that regulate gene expression without changing the underlying DNA sequence. A prominent example of epigenetic modification is DNA methylation, which silences genes that are critical for T cell activity. Ghoneim et al. identified the whole-genome de novo DNA methylation program by the enzyme DNMT3A, which promotes the terminal differentiation of exhausted T cells, and revealed that these programs remain active even after anti-PD-1 therapy.^314^ Histone modifications also contribute significantly to the exhausted state of T cells. Specific alterations, such as increased histone acetylation and methylation, affect chromatin structure and accessibility, influencing gene expression. A study in 2022 confirmed that tumor cells competitively inhibit methionine utilization by CD8^+^ T cells by overexpressing SLC43A2 receptors. Methionine depletion leads to the downregulation of SAM and H3K79me2 in CD8^+^ T cells and affects T cell immunity through the STAT5 pathway.^315^

The epigenetic modifications observed in exhausted T cells, including changes in DNA and histone modifications and chromatin remodeling, contribute to the impaired function of these cells. Future research might focus on elucidating the role of epigenetic changes in T cell exhaustion. Understanding how chromatin remodeling and histone modifications contribute to the exhaustion phenotype could provide new therapeutic targets.

## Emerging therapies and challenges for reversing T cell exhaustion

As immunotherapy research progresses, many emerging therapies aimed at reversing T cell exhaustion have been developed (Table 1). Immune checkpoint inhibitors and cytokine-based therapies can directly target exhausted T cells. In contrast, strategies such as vaccines and CAR (Chimeric Antigen Receptor)-T cell therapies, while not directly addressing exhausted T cells, enhance immune responses by improving T cell function or providing additional immune stimulation. The overall enhancement of the immune response can partially reverse T cell exhaustion caused by factors such as high antigen load and immune evasion. Additionally, some pharmacological agents capable of modulating the immunosuppressive microenvironment (e.g., TGF-β inhibitors, A2aR inhibitors, CD73 inhibitors, and IDO1 inhibitors) can reduce the levels of immunosuppressive molecules within the microenvironment, such as TGF-β, adenosine, and kynurenine, thus enhancing T cell functionality and activity. These agents have the potential to act as valuable adjuncts to other primary immunotherapies, thereby facilitating a more robust synergistic therapeutic effect.

**Table 1:** Therapeutic targets and clinical research progress in T cell exhaustion

Disease	Therapies	Targets	FDA-Approved Drugs	Start Marketing Date	National Drug Code	Clinical Trials	With Combination (Clinical Trials)	Phase	Scope of Clinical Trials
Cancer/Tumorigenesis	ICI	PD-1	Pembrolizumab (Keytruda)	20190801	0006-3026-04	–	–	-	-
			Dostarlimab (Jemperli)	20210422	0173-0898-03	–	–	-	-
			Enlonstobart (SG001)	–	–	NCT06132217	Simmitinib (a-VEGFR2)	I/II	Advanced Solid Tumors
		PD-L1	Atezolizumab (Tecentriq)	20190308	50242-917-01	–	–	-	
			Cosibelimab-ipdl (Unloxcyt)	20241213	83444-301-10	–	–	-	
			Socazolimab	–	–	NCT06459687	–	III	Uterine Cervical Cancer
		CTLA-4	Ipilimumab (Yervoy)	20110325	0003-2328-22	–	–	-	-
			Tremelimumab (Imjudo)	20221021	0310-4535-30	–	–	-	-
			Porustobart (HBM4003)	–	–	NCT05167071	–	I	Neuroendocrine Neoplasm and other solid tumors
		TIM-3	Sabatolimab (MBG453)	–	–	NCT02608268	PDR001 (a-PD-1)	I-Ib/II	Advanced malignancies
			Cobolimab (TSR-022)	–	–	NCT03680508	TSR-042 (a-PD-1)	II	Advanced Hepatocellular Carcinoma
			LY-3321367	–	–	NCT03099109	LY3300054 (a-PD-L1)	Ia/ Ib	Advanced solid tumor
		LAG3	Relatlimab (BMS-986016)	–	–	NCT01968109	Single agent or in combination with Nivolumab(BMS-936558)	I/IIa	Advanced solid tumors
			Opdualag	20220318	0003-7125-11	–	–	-	-
			Tebotelimab (MGD013)	–	–	NCT04653038	–	I	Malignant Melanoma
		TIGIT	Tamgiblimab (IBI939)	–	–	NCT04672356	Sintilimab	I	Advanced Lung Cancer
			AK127	–	–	NCT05951608	AK112	Ib/II	Advanced Malignant Solid Neoplasm
			Domvanalimab (AB154)	–	–	NCT04736173	Zimberelimab (AB122)	II	Non-Small Cell Lung Cancer
		VISTA	CA-170	–	–	NCT02812875	–	I	Advanced solid tumors and lymphomas
			JNJ-61,610,588	–	–	NCT02671955	–	I	Advanced Cancer
		B7-H3	131I-omburtamab	–	–	NCT03275402	–	II/III	Neuroblastoma central nervous system/leptomeningeal metastases
			Obrindatamab (MGD009)	–	–	NCT03406949	MGA012 (a-PD-1)	I	Advanced solid tumors
			Enoblituzumab (MGA271)	–	–	NCT01391143	–	I	Refractory Cancer
	Cytokine therapy	IL-2	Aldesleukin (Proleukin)	20240715	73776-022-01	–	–	-	-
			Denileukin diftitox-cxdl (Lymphir)	20250101	52658-7777-1	–	–	-	-
			Tucotuzumab (huKS-IL2)	–	–	NCT00408967	–	II	Recurrent Ovarian Carcinoma
		IL-15	Anktiva (N-803)	20240506	81481-803-01	–	–	-	-
			NKTR-255	–	–	NCT05664217	–	II/III	Diffuse Large B-Cell Lymphoma
			SHR-1501	–	–	NCT05410730	Single agent or in combination with BCG (Bacillus Calmette-Guérin)	I/II	Non-Muscle Invasive Bladder Neoplasms
		IL-12	NHS-IL12	–	–	NCT01417546	–	I	Metastatic Solid Tumors
			AS1409 (huBC1-IL12)	–	–	NCT00625768	–	I	Metastatic Renal Cell Carcinoma or Metastatic Malignant Melanoma
			Ad-RTS-hIL-12 and veledimex	–	–	NCT04006119	Cemiplimab-rwlc	II	Recurrent or Progressive Glioblastoma
		IL-7	Efineptakin alfa (NT-17)	–	–	NCT04984811	Atezolizumab	II	Advanced or Metastatic Non-Small Cell Lung Cancer
			MDK-703	-	-	NCT05716295	-	I/II	Advanced or Metastatic Solid Tumors
			BNT152	–	–	NCT04710043	BNT153 (a- IL-2)	I	Solid Tumors
		IL-21	BMS-982470	–	–	NCT01629758	BMS-936558 (a-PD-1)	I	Solid Tumors
			AMG 256	–	–	NCT04362748	–	I	Advanced Solid Tumors
			JS014	–	–	NCT05296772	Single agent or in combination with pembrolizumab	I	Advanced Cancer
	TGF-β inhibitor	TGF-β	Nisevokitug	–	–	NCT05417386	FOLFIRINOX	III	Metastatic Pancreatic Ductal Adenocarci
			Trabedersen	–	–	NCT06079346	Single agent or in combination with FOLFIRINOX	III	Pancreatic Ductal Adenocarcinoma
			Galunisertib (LY2157299)	–	–	NCT03470350	Capecitabine	I/II	Activated Colorectal Cancer
	A2aR inhibitor	A2a receptor	PORT-6 (TT-10)	–	–	NCT04969315	–	II	Advanced Renal Cell Carcinoma
			Ciforadenant	–	–	NCT05501054	Ipilimumab、Nivolumab	I/II	Advanced Renal Cell Carcinoma
	CD73 inhibitor	CD73	Oleclumab (Anti-CD73 MAb)	–	–	NCT05221840	Oleclumab/durvalumab	III	Locally Advanced Lung Non-Small Cell Cancer
			Quemliclustat	–	–	NCT06608927	–	III	Metastatic Pancreatic Ductal Adenocarcinoma
			JAB-BX102	–	–	NCT05174585	Single agent or in combination with Pembrolizumab	I/II	Advanced Malignant Solid Neoplasm
	CD38 inhibitor	CD38	Daratumumab	20151116	57894-502-05	–		-	-
			Isatuximab-IRFC	20200302	0024-0656-01	–	–	-	-
			Erzotabart	–	–	NCT04824794	–	II	Diffuse large B-cell lymphoma recurrent
	IDO1 inhibitor	IDO1	Indoximod	–	–	NCT02835729	Idarubicin and Cytarabine	I	Acute Myeloid Leukemia
			Navoximod (GDC-0919)	–	–	NCT02471846	Single agent or in combination with Atezolizumab	Ib	Locally Advanced or Metastatic Solid Tumors
			Epacadostat	–	–	NCT02752074	Single agent or in combination with pembrolizumab	III	Unresectable or Metastatic Melanoma
Chronic infection	ICI	PD-1	Pembrolizumab (MK3475)	–	–	NCT02595866	–	I	HIV infection
		PD-L1	ASC22	–	–	NCT05330143	–	II	HIV infection
		CTLA-4	Ipilimumab	–	–	NCT03407105	–	I	HIV-infection
	Cytokine therapy	IL-15	Anktiva (N-803)	–	–	NCT02191098	–	I	HIV-infection

Notably, costimulatory agonist antibodies are a class of therapeutic agents that enhance T cell functionality by activating costimulatory molecules on T cell surfaces, such as CD28, CD137, OX40, and GITR.^316,317^ These antibodies have demonstrated promising potential in the immunotherapy of cancer and chronic infections. However, as previously mentioned, the lack of appropriate costimulatory signals more frequently leads to T cell anergy rather than exhaustion. Furthermore, excessive costimulation may exacerbate T cell exhaustion. Therefore, achieving a balance between T cell activation and exhaustion when these therapies are utilized presents a considerable challenge in clinical practice.^316^ Consequently, in the section discussing therapies for reversing T cell exhaustion, we do not provide a detailed examination of costimulatory agonist antibodies.

### Directly targeting exhausted T cells

#### Immune checkpoint inhibitors

ICIs represent a groundbreaking approach in cancer therapy, particularly in reversing T cell exhaustion.^318^ ICIs work by blocking specific receptors on T cells that inhibit their activity, rejuvenating T cell responses against tumors. A well-studied example of an ICI is anti-PD-1 therapy.^319^ Inhibiting PD-1 with antibodies, such as pembrolizumab (Keytruda) and nivolumab (Opdivo), reinvigorates exhausted T cells, enhances antitumor responses, and has made good progress in clinical treatment. Pembrolizumab and nivolumab have shown pronounced efficacy in treating metastatic melanoma.^320,321^ Compared with traditional therapies, these agents have significantly improved overall survival rates. Additionally, the use of pembrolizumab has transformed the treatment landscape for NSCLC. In patients with high PD-L1 expression, pembrolizumab has achieved a 44% response rate,^322^ demonstrating the significance of PD-L1 as a biomarker for treatment effectiveness. This highlights the role of T cell activation in inducing cancer regression. In chronic viral infections, ICIs are also being explored. For example, studies using anti-PD-1 therapy in patients with chronic HBV infections have demonstrated promising results.^323–325^ In these cases, blocking PD-1 can enhance the functionality of exhausted T cells, leading to improved viral control and potential viral eradication.

Despite the transformative impact of ICIs, several challenges remain. First, the TME can be immunosuppressive, harboring factors that facilitate T cell exhaustion. For example, in pancreatic cancer, the dense fibrotic stroma and the presence of immunosuppressive cytokines such as TGF-β severely limit the effectiveness of PD-1 inhibitors.^326,327^ This highlights the need for combination therapies that modify the TME.^328,329^ These findings emphasize the critical need for combination therapies designed to modulate the tumor microenvironment. Several potential therapeutic strategies targeting the TME include (1) disrupting the CXCR4‒CXCL12 axis to increase T cell infiltration into cold tumors;^330–332^ (2) employing JAK/STAT inhibitors to reduce the accumulation of tumor-associated myeloid cells;^333,334^ and (3) targeting Tregs through antibodies directed against CD25 or other relevant surface antigens.^335,336^ These approaches aim to reshape the TME to promote effective antitumor immunity and improve therapeutic outcomes.

Second, not all patients benefit equally from ICIs. Factors such as tumor mutational burden (TMB) influence patient response. For example, patients with high TMB usually exhibit better outcomes owing to the presence of more neoantigens, which stimulate T cell activity. In contrast, patients with a lower TMB may continue to experience T cell exhaustion despite treatment.^337–339^ Targeting DNA repair mechanisms or introducing DNA-modifying agents can increase the mutational burden within cancer cells.^340–342^ This strategy aims to exploit the increased genetic instability in tumor cells, promoting the presentation of immunogenic peptides and facilitating the activation of the immune system to recognize and eliminate cancerous cells more effectively.

Moreover, ICIs may provide an initial response in certain patients, but disease progression can occur over time. The development of acquired resistance is associated with increased expression of alternative checkpoints (e.g., TIM3 and LAG-3)^343,344^ or alterations in the TME that promote immune evasion;^345^ for example, resistance to PD-1 therapy in melanoma has been linked to gene mutations and changes in the TME that promote T cell dysfunction.^346,347^ Zaretsky et al. performed whole-exome sequencing on paired biopsy samples from baseline and relapsing lesions of four patients with metastatic melanoma.^347^ In two of these patients, resistance-related functional deletions were observed in Janus kinase 1 (JAK1) and Janus kinase 2 (JAK2), genes that play critical roles in interferon receptor signaling. Truncations in JAK1 and JAK2 fail to respond to INF-γ, leading to a loss of its anti-proliferative effects on melanoma cells. In a third patient, a truncating mutation in the beta-2-microglobulin (B2M) gene was detected, causing the absence of surface expression of MHC-I, which is crucial for antigen presentation.

Finally, T cell activation causes autoimmune-like side effects, affecting various organs, such as the gastrointestinal tract, skin, and endocrine system. The management of these immune-related adverse events (irAEs) poses a significant hurdle, as they impact the overall treatment strategy and patient quality of life.^348,349^ ICIs exert systemic effects rather than being localized to specific sites, such as infected tissues or cancers, enabling them to induce autoimmune damage across various tissues.^350–352^ For example, when they act on blood vessels, activated T cells may increase the burden of atherosclerosis and the instability of plaques; when they target the heart, T cell-mediated myocarditis may be induced.^353^ Epidemiological data suggest that approximately 8% of over 21,000 patients receiving ICI treatment experience cardiovascular events, with a mortality rate as high as 50% in those who develop myocarditis.^354,355^ The irAEs are classified on the basis of the Common Terminology Criteria for Adverse Events (CTCAE), with severity levels ranging from mild (grades 1–2) to severe (grades 3–4), and in some cases, they can be fatal (grade 5).^355,356^ Although they share similarities with autoimmune disorders, irAEs are typically characterized by a more rapid onset and greater severity. Fortunately, with prompt and appropriate intervention, the risk of chronic complications can generally be avoided.^357^ Current management guidelines for irAEs recommend discontinuation of ICI therapy, initiation of corticosteroids for grade 2 irAEs, and the immediate use of immunosuppressants (such as TNF inhibitors,^358–364^ interleukin-6 blockers,^365–367^ JAK inhibitors,^368–370^ and mTOR inhibitors^371–373^) for grade 3 or higher irAEs to prevent death and chronic progression.^374,375^

In summary, ICIs have dramatically changed the landscape of cancer therapy by effectively reversing T cell exhaustion and enhancing antitumor immunity, and their potential to provide durable responses has been demonstrated in melanoma, NSCLC, and HCC. However, challenges such as the immunosuppressive TME, variability in patient responses, acquired resistance, and irAEs necessitate ongoing research. Addressing these shortcomings is crucial for optimizing ICI therapies and improving outcomes for patients with cancer.

#### Cytokine therapy

Cytokines are signaling molecules that play vital roles in regulating immune responses. They can modulate T cell activity, promote survival, and restore effector functions in exhausted T cells. For example, studies have reported that exhausted T cells exhibit reduced expression of CD122 (IL-2/15 receptor beta chain) and CD127 (IL-7 receptor alpha chain), which hinders their homeostatic renewal and functional maintenance mediated by IL-7 and IL-15.^376^ Cytokine therapy aims to rejuvenate exhausted T cells by providing the essential signals for activation and proliferation.^377^ Cytokines are signaling molecules that play vital roles in regulating immune responses. They can modulate T cell activity, promote survival, and restore effector functions in exhausted T cells. Specific cytokines, such as IL-2, IL-7, and IL-15, have been explored for their potential to restore T cell functionality.

IL-2 is a key growth factor for T cells, as it enhances their proliferation and activation. High-dose IL-2 therapy has demonstrated effectiveness in certain cancers, such as melanoma and renal cell carcinoma.^378,379^ However, its short half-life and side effects, such as vascular leak syndrome, have restricted its use.^380^ These adverse effects underscore the importance of optimizing the dose to restore T cell functions without triggering excessive activation, which can induce toxicity. For example, a study showed that low-dose IL-2 therapy in a patient-derived melanoma organoid model improved the proliferation and functionality of CD4^+^ and CD8^+^ T cells, leading to enhanced antitumor responses.^381^

IL-7 plays a critical role in T cell homeostasis and survival. It reportedly improves the survival of memory T cells and promotes the re-expansion of T cell populations following activation.^382^ In a chronic LCMV infection mouse model, IL-7 therapy successfully rejuvenated exhausted T cells during chronic viral infections.^383^ This rejuvenation led to improved control of the virus and enhanced antiviral immunity. Moreover, Koyas et al. reported that the signal transduction of IL-7 counteracted the immunosuppressive effect of adenosine on tumor-infiltrating T cells, improving the antitumor immune response in melanoma models.^384^

IL-15 has gained attention for its capacity to stimulate the proliferation of memory T cells without inducing T cell activation-induced cell death. IL-15 is essential for the proliferation and survival of memory CD8^+^ T and NK cells.^385^ In preclinical studies and early-phase clinical investigations, IL-15 has shown promise in reinvigorating exhausted T cells in cancer therapies. Combining IL-15 with checkpoint inhibitors has demonstrated synergistic effects, resulting in enhanced antitumor immunity.^386,387^ In 2024, Anktiva, an IL-15 super agonist drug for treating solid tumors, hematological cancers, and HIV infection, was approved for marketing, becoming the world’s first IL-15 drug approved and the third cytokine drug to be used for tumor immunotherapy.^388^ Anktiva comprises an IL-15 N72D mutant that binds to the IL-15R α sushi domain and the IgG1 Fc fusion protein. The IL-15R α sushi domain eliminates the essential trans presentation of natural IL-15 to activate downstream signaling pathways, and the IgG1 Fc fragment greatly prolongs the drug’s half-life.

**Fig. 4:**
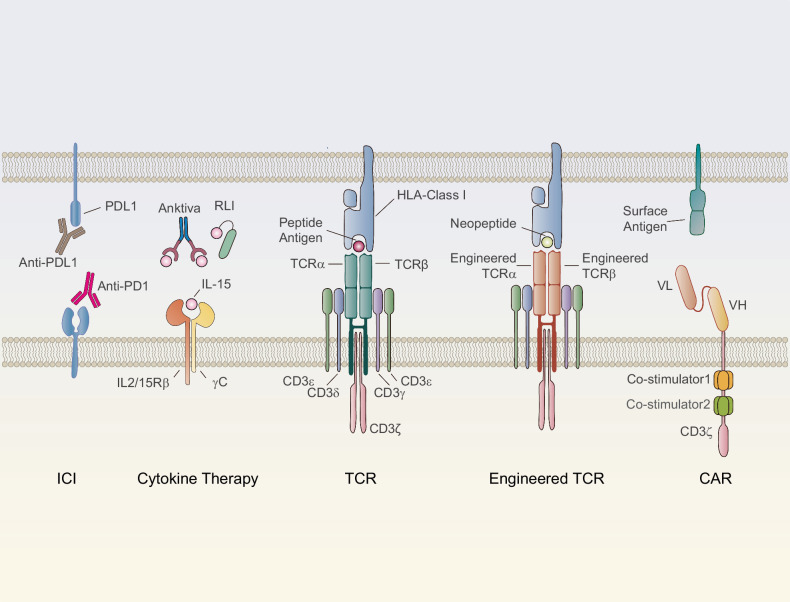
T cell surface receptor-based activation strategies for enhanced immune responses. An approach involves the genetic engineering of TCRs to increase the specificity and precision in targeting tumor-associated antigens. Engineered TCRs are designed to recognize and bind tumor-specific epitopes, improving tumor targeting while minimizing off-target effects. An alternative strategy introduces MHC-independent chimeric antigen receptors, which bypass antigen presentation by MHC molecules, enabling T cells to directly target tumor cells on the basis of surface markers. Another crucial component of these strategies is the targeting of immune checkpoint receptors (e.g., PD-1 and CTLA-4), which can be blocked to prevent T cell exhaustion, sustain T cell activity and improve antitumor responses. Additionally, cytokines can be used to further enhance T cell expansion, persistence, and functionality. Engineered cytokines, such as IL-15, stimulate T cell proliferation, survival, and effector function, increasing the overall immune response

While cytokine therapies have shown promise in reversing T cell exhaustion, challenges remain in optimizing their use in clinical settings. First, the use of high-dose cytokines usually leads to severe side effects, including fever, fatigue, cytokine storm,^389^ and organ toxicity.^390^ This risk necessitates careful monitoring and management during treatment. For example, high-dose IL-2 causes vascular leak syndrome, leading to hypotension and organ dysfunction.^380^ These adverse effects limit dose escalation and, consequently the efficacy of the therapy. To mitigate off-target and side effects, targeted antibodies can be paired with engineered cytokine mutants that exhibit reduced receptor affinity, thereby minimizing undesired interactions between cytokines and nontarget cells. Orionis Biosciences is advancing a novel class of immunocytokines termed activity-on-target cytokines (AcTakines)^391^ (Fig. 4). These engineered molecules feature a combination of a targeting antibody and a cytokine mutant with decreased receptor affinity. This reduction in affinity effectively prevents premature cytokine activity, allowing the fusion protein to accumulate selectively at the target cell, where it can bind to the receptor and activate its therapeutic effects, thereby significantly reducing off-target effects.

Second, cytokines can be sequestered by their cognate receptors in the circulation before their intended cells are targeted, a phenomenon known as the “cytokine sink” effect.^392,393^ For example, the dimeric IL-2Rβγ complex is expressed primarily on CD4^+^ and CD8^+^ memory T cells, as well as NK cells, which have relatively low IL-2Rα expression.^394^ In contrast, the high-affinity trimeric IL-2Rαβγ complex is predominantly found on regulatory T cells,^394^ where its activation can lead to immunosuppression, counteracting the desired immune activation in immunotherapies. Protein-directed evolution can be applied to generate IL-2 mutants with enhanced affinity for the IL-2Rβγ complex.^395,396^ The process of engineering these muteins involves error-prone PCR to generate a mutagenic IL-2 library, which is then screened for variants with improved IL-2Rβ affinity. This results in the identification of an IL-2 “superkine” with a 200-fold increase in affinity for IL-2Rβ.^396^ Superkine effectively reduces the affinity gap between dimeric and trimeric receptor complexes, leading to enhanced antitumor efficacy and minimized toxicity in mouse models.

Cytokine therapy represents a compelling strategy to reverse T cell exhaustion, enhancing immune responses against chronic infections and cancers. As ongoing research continues to investigate the mechanisms and effects of cytokines on T cell functionality, these therapies could significantly improve patient outcomes in cancer and chronic disease management. However, the associated side effects, variability in patient responses, and transient effects of treatment underscore the need for further research. The dosage, timing, and specific context of therapy need careful consideration to avoid potential adverse effects.

### Overall immune response enhancement

#### CAR-T cEll Therapy

CAR-T-cell therapy involves harvesting T cells from a patient and genetically engineering these cells to express CARs that recognize specific tumor antigens.^397,398^ The CAR comprises an extracellular region that identifies a tumor-specific antigen, a transmembrane section, and an intracellular signaling domain that activates T cell function upon recognition of the antigen (Fig. 4). These receptors enable T cells to recognize specific tumor-associated antigens (TAAs) independent of the MHC, directly targeting malignant cells. After engineering, engineered T cells are cultured and proliferate in the laboratory to generate a sufficient number of T cells for therapeutic use. Once reintroduced into a patient, CAR-T cells identify and attach to the TAA present on tumor cells, inducing T cell activation. The engineered CAR provides a more robust activation signal, promoting T cell proliferation and persistence in the TME; thus, it can overcome some of the limitations related to traditional T cell responses, including exhaustion.

A notable example of CAR-T cell therapy is Kymriah (tisagenlecleucel), which is approved by the FDA for treating acute lymphoblastic leukemia (ALL) in pediatric and young adult patients.^399^ Kymriah targets the CD19 antigen, which is commonly expressed on B-cell malignancies. In clinical trials, Kymriah reported a profound overall remission rate of approximately 83% in patients with recurrent or treatment-resistant ALL, indicating the potential of CAR-T cell therapy to achieve significant clinical outcomes.^399,400^ Another FDA-approved CAR-T product, axicabtagene ciloleucel (Yescarta), also targets CD19 and has shown significant success in patients with diffuse large B-cell lymphoma (DLBCL).^401,402^ In pivotal studies, the overall response rate in patients was approximately 80%, with 60% achieving complete remission, highlighting the capacity of CAR-T therapy to reactivate T cells against aggressive lymphomas.^401^

CAR-T cell therapy has emerged as a groundbreaking approach for certain cancers, particularly hematological malignancies. However, despite its success, CAR-T cell therapy presents several challenges that limit its broader application. First, although CAR-T cells initially restore T cell function, many patients experience a decrease in CAR-T cell persistence over time, leading to disease recurrence. For example, although the early response rates in B-ALL patients are considerable, relapse occurs in many patients, which is usually attributed to the loss of CAR-T cell function and persistence.^399^ Moreover, tumors undergo genetic changes that cause the loss of the target antigen, resulting in treatment resistance.^403^ In cases where CD19-targeted CAR-T cells are used, some relapsed patients have CD19-negative leukemic cells, leading to treatment failure.^399,404,405^ This highlights the need for multitarget CAR designs to prevent tumor escape. The incorporation of additional costimulatory domains, such as 4-1BB (CD137) or OX40,^406–408^ into CAR constructs can improve T cell survival and functional longevity. These domains provide essential second signals during T cell activation, promoting enhanced proliferation and persistence even in the presence of inhibitory signals. Additionally, culturing CAR-T cells with IL-7 and IL-15 supports T cell survival and expansion after infusion, resulting in greater antitumor activity.^409,410^

Second, the swift proliferation of CAR-T cells causes cytokine release syndrome (CRS),^411–413^ a serious health risk characterized by profound cytokine release.^414^ To standardize the grading of CRS globally, the American Society of Blood and Marrow Transplantation (ASBMT) has published consensus guidelines. These guidelines outline fever as an essential diagnostic criterion, with the severity of CRS being primarily determined by the presence and extent of hypotension and hypoxia.^415^

In clinical trials, CRS was observed in as many as 93% of individuals with large B cell lymphoma in the ZUMA-1 trial, with severe cases requiring intensive management.^402^ A cohort of 68 pediatric and young adult individuals diagnosed with relapsed or refractory ALL received treatment with Kymriah. Among these patients, 79% experienced CRS, with 49% classified as having a grade ≥ 3 CRS.^399^ This side effect limits the application of CAR-T therapy in patients who may not tolerate aggressive immune activation. Clinical evidence supports a direct correlation between IL-6 levels and the severity of CRS in patients receiving CAR-T cell therapy.^399,402^ An anti-IL-6 receptor antagonist, tocilizumab, has demonstrated significant efficacy in controlling CRS and was approved by the FDA for this purpose in 2017.^416–420^ In addition, corticosteroids have proven effective in managing treatment-related toxicities.^416^ For patients who show an inadequate response to IL-6 receptor blockade, prompt administration of corticosteroids is recommended. In severe cases, particularly those with grade 3 or higher CRS, rapid clinical deterioration, and unstable vital signs, intensive care unit admission is necessary for appropriate stabilization and management.^402,412,416^

Third, while CAR-T cell therapy has shown profound efficacy in treating hematological malignancies, its implementation in solid tumors is challenging. In contrast to hematological cancers, which usually express unique and targetable antigens, solid tumors frequently exhibit a diverse array of tumor-associated antigens with high heterogeneity in different cancer cells.^421^ Additionally, these antigens are expressed at low levels in normal tissues.^422^ This heterogeneity complicates the effective targeting of tumor cells, as CAR-T cells may fail to recognize or eliminate all tumor variants, causing tumor relapse or progression. Additionally, in contrast to the relatively supportive environments in hematological malignancies, solid tumors usually create hostile microenvironments characterized by hypoxia, nutrient deprivation, and the presence of immunosuppressive factors. Therefore, the development of CAR-T cell therapies targeting solid tumors necessitates the incorporation of advanced strategies such as genomics and proteomics to identify highly specific and diverse antigens that are uniquely expressed on cancer cells.^423–428^ This approach aims to increase the specificity and effectiveness of CAR-T cell recognition of optimal target antigens. Furthermore, it is crucial to design strategies that address the challenges posed by the immunosuppressive and hypoxic tumor microenvironment,^429,430^ which hinders CAR-T cell efficacy. Other strategies, such as promoting T cell infiltration into tumor sites^431–433^ and mitigating T cell exhaustion,^434–436^ are critical for improving therapeutic outcomes. Here, we briefly introduce several strategies to improve CAR-T cell exhaustion at the CAR design level. One key issue is self-aggregation of the CAR, which leads to tonic signaling.^437^ This aggregation, driven by the scFv region, results in ligand-independent activation that can impair CAR-T cell function, contributing to exhaustion. Self-aggregation is predominantly influenced by the framework region (FR) of the scFv rather than the complementary determining region (CDR).^437,438^ To mitigate this, one promising approach is the grafting of the CDR into the FR of a nonaggregating scFv, which can help prevent undesired aggregation.^434^ This strategy has been successfully applied in the development of hybrid scFv constructs, such as those combining the CDR from the GD2 CAR and the FR from the CD19 CAR, reducing CAR exhaustion.^435^

Furthermore, the replacement of the CD28 intracellular domain with that of 4-1BB in the GD2 CAR construct has been shown to generate a less potent costimulatory signal. The weaker signaling from the 4-1BB domain appears to be sufficient for effective T cell activation without promoting the exhaustion phenotype associated with sustained CD28-driven signaling.^435^ This modification leads to reduced exhaustion markers, enhanced cytokine production in vitro, and improved T cell persistence in vivo.

Additionally, various modifications to the CAR structure can be employed to mitigate tonic signaling. One such modification involves shortening the VH sequence and replacing the Ig-derived hinge and CD28 transmembrane domains with those from human CD8α in the anti-GD2 CAR.^439^ This alteration reduces self-dimerization and decreases the expression of exhaustion markers such as PD-1. Moreover, the spacer region between the scFv and the transmembrane domain plays a crucial role in modulating tonic signaling. Notably, CARs incorporating only the CH3 domain of the immunoglobulin, rather than both CH3 and CH2, have demonstrated a reduction in ligand-independent signaling activation, thereby preventing exhaustion.^440^

Another approach to halt tonic CAR signaling is to regulate CAR expression dynamically, turning it off when not required.^436^ One promising strategy involves incorporating a destabilizing domain (DD) into the CAR construct.^441^ In this system, CAR expression is halted via degradation, but the application of a stabilizing agent can counteract this effect, preventing degradation and restoring CAR expression. This method allows for the potential recovery of a memory-like phenotype in exhausted CAR T cells by reactivating CAR expression-T cells, thus supporting the concept that CAR-induced exhaustion is a reversible process.

Finally, the complex and lengthy procedure of CAR-T cell production poses challenges for its common use. Each treatment requires individualized T cell harvesting, genetic modification, and expansion, which require several weeks. This delay may be detrimental in aggressive malignancies where timely treatment is critical. This has prompted the development of universal CAR-T cell therapies to simplify manufacturing processes, reduce costs, improve patient access to this promising treatment, and create a more accessible and versatile treatment option.^442^

Universal CAR-T cell therapy refers to the generation of genetically modified T cells that can be used for many patients without customization for each individual, making the therapy more accessible.^442^ This is achieved through the use of an “off-the-shelf” approach, where T cells are derived from healthy donors by removing or modifying the genes that may cause rejection (e.g., the T-cell receptor). These cells are less likely to trigger an adverse immune response in the recipient.

Several recent developments in universal CAR-T cell therapies have garnered significant attention. The utilization of CRISPR/Cas9 for gene editing has facilitated the development of these therapies by allowing the creation of T cells with specific targets while minimizing the risk of rejection. Researchers have explored allogeneic versions of CART-19 that target CD19-positive malignancies. CRISPR Therapeutics evaluated the effectiveness and safety of allogeneic CAR-T cell therapy CTX110^TM^ for CD19^+^ B cell malignancies. Preliminary results revealed that in patients with large B-cell lymphoma, high-dose CTX110 monotherapy resulted in an objective response rate (ORR) of 58% and a complete response (CR) rate of 38%, with good safety.^443^ B-cell maturation antigen (BCMA) is considered a potential target in multiple myeloma. Companies such as Allogene Therapeutics and Celyad Oncology have developed allogeneic CAR-T cells that target BCMA, demonstrating potential in preclinical and early clinical studies.^444,445^ Pan et al. recently reported the results of a phase 1 trial of allogeneic CD5-specific CAR-T therapy for the treatment of relapsed/refractory T-ALL. The results indicated that this CD5-specific CAR-T intervention had a high response rate for patients with T-ALL and that combined transplantation can reduce the risk of delayed severe infections.^446^

Despite promising developments in universal CAR-T therapies, several challenges remain.^447^ The recipient’s immune system may pose a risk of rejection of the transplanted allogeneic CAR-T cells.^448^ Although modifications reduce the risk of host-versus-graft rejection (HvGR) and graft-versus-host disease (GVHD),^448,449^ this remains a significant concern. Strategies to mitigate this risk are critical for the successful implementation of universal CAR-T therapies. Similarly, while early results are promising, the safety and sustained effectiveness of universal CAR-T therapies should be established through rigorous clinical trials. Moreover, similar to innovative therapies, navigating regulatory pathways and obtaining approval for universal CAR-T products can be complex and time-consuming.

In summary, CAR-T cell therapy is a cutting-edge approach to cancer treatment that combines advanced genetic engineering with immunotherapy to recognize and kill cancer cells. Successes in ALL and DLBCL exemplify its potential. However, limitations such as T cell longevity, CRS, restricted effectiveness in solid tumors, antigen escape, and manufacturing challenges should be addressed. As ongoing research continues to refine CAR designs and rigorous quality control measures are used to ensure the safety and functionality of CAR-T cells, CAR-T cell therapy holds great promise for improving outcomes in various cancers.

#### Vaccine strategies to disrupt exhaustion status

**Fig. 5:**
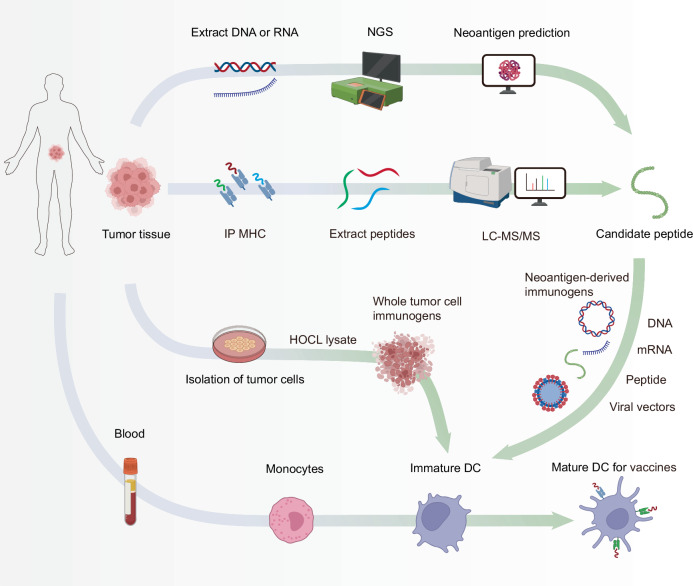
Methods for tumor neoantigen discovery and vaccine design strategies Neoantigen discovery begins with the identification of somatic mutations in tumor cells, followed by the prediction of potential neoantigens via bioinformatics tools for analyzing tumor-specific mutations and their binding affinity to host MHC molecules. The peptides are subsequently synthesized and validated through techniques such as mass spectrometry or T cell assays. An alternative approach for neoantigen discovery involves the immunoprecipitation of MHC molecules from tumor tissues, followed by acid elution of bound peptides and subsequent identification through de novo mass spectrometry sequencing. For neoantigen presentation, various delivery strategies have been developed, including peptide-based vaccines, DNA or mRNA encoding the neoantigens, and viral vector systems. Additionally, neoantigens can be used in dendritic cell (DC) vaccines, as loading with neoantigens or tumor lysates can activate DCs for efficient presentation to T cells. These approaches aim to enhance antitumor immunity by specifically targeting tumor cells with minimal off-target effects

**Fig. 6:**
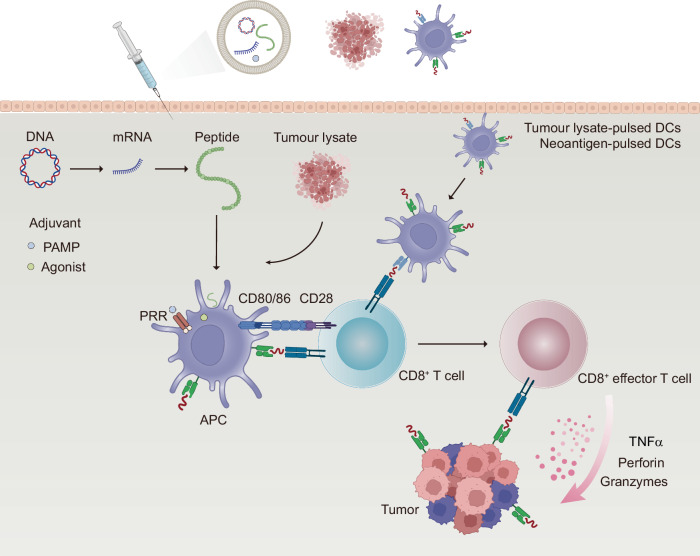
Mechanisms of neoantigen-based therapeutic cancer vaccines. Upon injection, the neoantigens, in peptide form, are taken up by antigen-presenting cells, such as DCs, which handle and present the antigens via MHC molecules. These activated APCs reach the lymph nodes where they initiate and induce T cell maturation, which is subsequently directed to target and destroy tumor cells. Alternatively, mature DCs preloaded with tumor-specific neoantigens can be directly administered to facilitate T cell activation and antitumor immune responses. Moreover, adjuvants activate DCs through pathogen-associated molecular patterns (PAMPs) or enhance immune responses through various immune protein agonists

Innovative strategies focused on specific antigens and vaccine designs are emerging to reinvigorate T cells and boost antitumor immunity^450,451^ (Figs. 5, 6). Early therapeutic vaccination strategies primarily target TAAs, which are self-antigens abnormally expressed or overexpressed in tumors. However, these approaches have usually been clinically unsuccessful owing to central and peripheral tolerance mechanisms.^452^ Moreover, TAA expression in noncancerous tissues highlights the potential for autoimmune toxicity following vaccination. In contrast, mutations in tumor cells generate novel self-antigenic epitopes known as neoepitopes or neoantigens. Vaccines based on neoantigens offer several advantages over traditional TAA-based vaccines. First, neoantigens are expressed only by tumor cells, eliciting a targeted tumor-specific T cell response that minimizes off-target damage to healthy tissues. Second, because neoantigens arise from somatic mutations, they circumvent the central tolerance mechanisms that prevent T cells from reacting to self-epitopes, promoting robust immune responses against tumors. Additionally, the enhanced neoantigen-specific T cell response elicited by these vaccines establishes posttreatment immune memory, offering a promising avenue for long-term prevention of disease recurrence. Personalized neoantigen vaccines that target unique mutations in an individual’s tumor have shown potential in enhancing T cell responses. Common vaccine types that target neoantigens include peptide, viral, DNA, and mRNA vaccines.

In addition to neoantigens, whole tumor cell (WTC) vaccines represent a viable and encouraging therapeutic approach^453,454^ (Fig. 6). Whole tumor cell vaccines use entire tumor cells, live or killed, as the basis for stimulating the immune system. They present various TAAs, including common and tumor-specific antigens, to the immune system. These antigens exist on tumor cell surfaces and activate innate and adaptive immune responses, including those involving macrophages, DCs, and T cells. To prepare whole tumor cell vaccines, tumor cells are usually harvested from patients or established cell lines and subjected to processes such as irradiation, heat, or chemical treatments to render them nonviable, ensuring that they do not cause tumor formation upon injection. These inactivated tumor cells are subsequently injected directly into the patient or delivered alongside adjuvants to enhance the immune response.

DCs are pivotal in T cell activation and can be utilized in vaccine strategies to enhance T cell function. By providing proper signals for T cell activation, DC vaccines help combat T cell exhaustion. Vaccines that involve loading DCs with whole-tumor lysates or neoantigens effectively stimulate T cell responses. For example, a DC vaccine loaded with the gp100 peptide has been tested in patients with melanoma, resulting in increased T cell proliferation and improved clinical outcomes.^170^

Furthermore, adjuvants play a key role in shaping the immune response and preventing T cell exhaustion. For example, the inclusion of toll-like receptor (TLR) agonists in vaccine formulations reportedly enhances the effectiveness of T cell responses. In a study involving a TLR-3 agonist combined with a melanoma vaccine, T cell activation and proliferation were enhanced, which helped overcome the exhaustion phenotype.^455,456^ Additionally, the combination of STING agonists and IL-12 mRNA delivered by LNPs (lipid nanoparticle delivery) leads to better clinical outcomes, indicating promising novel progress in cancer immunotherapy.^457^ Notably, the choice of adjuvant greatly influences the outcome. Certain adjuvants may induce overactivation of immune responses, causing adverse effects or toxicity, whereas others may fail to generate a robust response. The optimization of adjuvant formulations is crucial but complicates the development of effective vaccine therapies. Chen et al. introduced the cytoplasmic membrane of *Escherichia coli* into tumor membrane antigen vaccines, which simultaneously present tumor antigens and adjuvants to DCs to trigger strong innate and tumor-specific adaptive immune responses.^458^ This approach effectively reduced tumor recurrence, improved survival rates in tumor-bearing mice, and provided long-term, tumor-specific immunity upon rechallenge.

Despite their potential benefits, vaccine strategies aimed at reversing T cell exhaustion have several limitations.^459–461^ First, tumors can evolve and lose the expression of targeted antigens, causing treatment resistance. This highlights the need for vaccines targeting multiple antigens to mitigate the risk of antigen escape. Second, the vaccination timing relative to the disease stage can significantly impact treatment efficacy. The vaccination of patients at the later stages of cancer or during periods of heightened immune suppression may diminish the effectiveness of vaccine strategies. Combining vaccines with other therapeutic modalities, such as ICIs or conventional therapies, may be essential; however, this combination increases the complexity of treatment regimens. Finally, the existence of immunosuppressive elements in the TME inhibits vaccine efficacy. Tumors usually secrete cytokines such as IL-10 and TGF-β, which contribute to T cell exhaustion and inhibit T cell function. Even with an effective vaccine, the immunosuppressive microenvironment prevents the full restoration of T cell activity. Similar to CAR-T therapy, these challenges highlight the importance of exploring highly specific and diverse tumor-associated antigens, as well as strategies for managing the tumor microenvironment.

The design and application of specific antigens and innovative vaccine strategies represent promising frontiers in reversing T cell exhaustion. However, challenges such as variable efficacy, an immune suppressive TME, antigen loss, adjuvant requirements, and intervention timing should be addressed to optimize the effectiveness of these therapeutic strategies, and continued research and clinical studies are essential to refine vaccine strategies and overcome the barriers associated with T cell exhaustion.

### Exploration of combination therapy

#### Checkpoint inhibitors and targeted therapies

Combining ICIs, such as PD-1 or CTLA-4 blockers, with targeted therapies aids in restoring T cell function through the modulation of the TME and the reduction of inhibitory signals. For example, in the treatment of metastatic melanoma, patients are treated with the PD-1 inhibitor pembrolizumab alongside the targeted therapy vemurafenib, which inhibits the BRAF oncogene.^462,463^ The results showed that this combination led to significant cancer regression and resulted in the downregulation of exhaustion markers on circulating T cells. The dual action of inhibiting tumor growth and the immune checkpoint enhances the overall effectiveness and persistence of the T cell response. Furthermore, combining atezolizumab (an anti-PD-L1 antibody) with bevacizumab (an anti-VEGF antibody) has been shown to improve outcomes in patients with HCC.^464^ The rationale behind this combination is that by blocking PD-L1, the inhibition of T cell responses is abolished, whereas bevacizumab reduces tumor hypoxia and improves T cell infiltration into the tumor. This dual approach addresses immune evasion by blocking PD-L1 and counteracting the immunosuppressive effects of VEGF, enhancing T cell responses.

#### Combination of checkpoint inhibition and vaccination

Combining checkpoint inhibitors with cancer vaccines aims to stimulate robust T cell responses while simultaneously removing the impediments that exhaustion imposes on T cell activity. A clinical trial investigated the use of the anti-PD-1 antibody nivolumab in combination with a personalized neoantigen vaccine in patients with solid tumors.^465^ The results indicated that all patients exhibited neoepitope-specific T cell responses after vaccination, and vaccine-induced T cells exhibited a cytotoxic phenotype with the ability to migrate to tumors and kill cells. Notably, T cells from patients receiving the combination therapy presented reduced exhaustion markers, suggesting that the vaccine primed T cells, whereas nivolumab prevented their functional inhibition. For example, in a previous study, combining a peptide vaccine targeting the MAGE-A3 antigen with anti-PD-1 blockade in patients with melanoma resulted in increased T cell activity and improved clinical outcomes.^466^ The addition of checkpoint blockade reinvigorated exhausted T cells, allowing them to activate a more effective response against tumor cells. This approach addresses the exhaustion status by enhancing T cell activation through the vaccine and overcoming the inhibitory signals mediated by checkpoints.

#### Synergistic combinations of vaccination and cell therapy

To address the challenges of the limited effectiveness of CAR-T cell therapy in solid tumors, this therapy has been combined with vaccines to enhance CAR-T responses and promote the immune system’s production of new T cells that target additional tumor antigens. In 2019, Ma et al. tested this approach by administering a vaccine carrying the same antigen targeted by CAR-T cells shortly after their infusion in a murine glioblastoma model, resulting in a significant increase in CAR-T cell effectiveness.^467^ Further research in 2023 revealed that this “vaccine + CAR-T” combination therapy significantly enhances DC recruitment to the TME, improving DC uptake of tumor antigens and activating endogenous antitumor T cells.^468^ While the results show promise, obstacles continue to exist, particularly in determining the optimal timing and coordination between vaccination and CAR-T therapy. A careful clinical trial design is needed to identify the optimum treatment schedules for maximizing efficacy. Additionally, combining these therapies may increase the risk of adverse effects, such as autoimmunity, making close monitoring for immune-related adverse events essential in clinical applications.

#### Dual-checkpoint inhibition

Using multiple checkpoint inhibitors to target different inhibitory pathways aids in overcoming T cell exhaustion more effectively than monotherapy does. The use of ipilimumab (Yervoy), an anti-CTLA-4 antibody, has shown significant promise in combination with PD-1 inhibitors to further enhance T cell activation and function. The combination of nivolumab and ipilimumab has demonstrated substantial efficacy in the treatment of metastatic melanoma.^321,469,470^ A landmark study involved assessing the coadministration of ipilimumab and nivolumab in patients with advanced melanoma; this combination therapy markedly improved overall survival compared with either agent alone.^470^ T cells from patients treated with both inhibitors presented decreased expression of exhaustion markers, suggesting that dual blockade synergistically reinvigorates T cell function and enhances clinical outcomes.

#### Metabolic intervention and immunotherapy

Combining metabolic interventions with immunotherapy is a promising strategy for reversing T cell exhaustion and enhancing antitumor immune responses. Combining metabolic modulation with immunotherapeutic approaches enhances T cell activity and restores T-cellT cell capacity to effectively combat tumors. For example, the effects of bezafibrate, a compound that activates PGC-1α/PPAR complexes in combination with anti-PD-1 therapy, were examined in cancer models.^471^ The results showed that bezafibrate activated the mitochondria of cytotoxic T lymphocytes, promoting oxidative phosphorylation and glycolysis. This metabolic reprogramming enhanced the expansion of naïve T cells and strengthened the effector functions of CTLs. Additionally, bezafibrate enhanced fatty acid oxidation and mitochondrial respiration, enabling stressed cells to meet heightened energy needs and supporting their survival. These results emphasize the critical importance of bezafibrate in maintaining a functional CTL population by stimulating mitochondrial and cellular metabolism, enhancing antitumor immunity during PD-1 blockade. Another metabolic intervention, the glutathione prodrug N-acetylcysteine (NAC), was shown to significantly impact glucose and lipid metabolism in CD8^+^ T cells in vitro. NAC promoted the differentiation of CD8^+^ T cells into long-lived memory T cells and enhanced TCF1 expression, which helped alleviate T cell exhaustion and apoptosis.^472,473^ In tumor-bearing mice, the combination of NAC with anti-PD-1 therapy inhibited colorectal cancer (CRC) progression.^474^ NAC facilitated the differentiation of TCF1^+^PD1^+^CD8^+^ T cells, decreasing the accumulation of exhausted T cells, suggesting a collaborative effect alongside PD-1 blockade.

The exploration of combination therapy for reversing T cell exhaustion has shown significant potential in improving the effectiveness of cancer immunotherapies. By leveraging various modalities, such as ICIs, targeted therapies, vaccinations, and immune modulators, researchers are discovering novel approaches to reinvigorate exhausted T cells and promote durable antitumor responses. However, combination therapies cause increased toxicity and complexity in treatment regimens. Identifying the optimal combinations and sequences of therapies remains challenging. Moreover, not all combinations have synergistic effects, leading to variable outcomes among patients. Continued investigations into these combination strategies are crucial for refining treatment regimens and improving outcomes in patients with cancer.

### IndicAtors For Evaluating The Recovery of T cell function

T cell function restoration is crucial for assessing immune recovery, particularly after immunotherapy, such as ICIs and CAR-T cell therapy. Monitoring T cell function involves a variety of markers that provide insights into immune activation, response to therapy, and overall immune reconstitution. The following markers are among the most widely employed to assess the restoration of T cell function in clinical settings.

1. One of the primary indicators of T cell function recovery is the peripheral blood CD8^+^ T cell count. The CD8^+^ T cell population is a critical component of the immune system’s ability to mount effective immune responses.^475^ A significant increase in the number of circulating CD8^+^ T cells, often observed after immunotherapeutic interventions, is a strong indicator of T-cell reconstitution and enhanced immune surveillance. Flow cytometry remains the gold standard for quantifying CD8^+^ T cells in peripheral blood, allowing for precise enumeration and characterization of these cells.
2. T cell activation markers are crucial for assessing the functional state of T cells. Cytokines such as IFN-γ, TNF-α, and IL-2 are produced by T cells upon activation and are considered key indicators of immune reactivation. Elevated levels of these cytokines are often observed following treatment with immune checkpoint inhibitors or other immune-stimulating therapies. The assessment of cytokine production can be performed via flow cytometry with intracellular cytokine staining or via enzyme-linked immunosorbent assays (ELISAs), both of which provide reliable quantification of T-cell activation.
3. T cell proliferation, assessed via Ki-67 expression, is another important marker.^476,477^ Ki-67 is a nuclear protein associated with cell proliferation and is widely used as a marker for active cell division.^476^ The upregulation of Ki-67 expression in CD8^+^ T cells indicates clonal expansion, a hallmark of the immune response during effective immunotherapy.^477,478^ The proliferation of T cells is crucial for maintaining long-term immune memory and enhancing antitumor immunity. Flow cytometry remains the primary method for assessing Ki-67 expression and evaluating T-cell proliferative capacity.
4. Immune cell subset analysis helps further evaluate T cell functionality. The ratio of naïve, memory, and effector T cells provides insights into immune quality.^475,479^ A greater proportion of effector memory CD8^+^ T cells often suggests restored immune function and the capacity for a rapid antigen response.^480^ This analysis is typically conducted via flow cytometry to assess immune cell phenotypes.
5. Immunosuppressive molecules such as TGF-β and IL-10 contribute to immune tolerance and suppression,^177,178^ particularly within the tumor microenvironment.^178^ A reduction in the levels of TGF-β and IL-10 following immunotherapy suggests a decrease in immune suppression and a concomitant restoration of T cell function. These cytokines can be detected in peripheral blood via ELISA, providing important markers of therapeutic efficacy and immune reactivation.
6. The expression of CD39 and CD73, enzymes involved in adenosine generation and immune suppression, can provide additional insight into T cell reactivation.^481–483^ These ectoenzymes contribute to the immunosuppressive environment by generating adenosine, which inhibits T cell function.^482,483^ The downregulation of CD39 and CD73 expression on immune cells following immunotherapy suggests a reduction in immune suppression, allowing for enhanced T-cell activity and function. Flow cytometry is commonly used to assess the expression of these markers in peripheral blood.
7. Circulating tumor DNA (ctDNA) is a valuable biomarker for monitoring T cell-mediated tumor clearance.^484–486^ As T cells target and eliminate tumor cells, the levels of ctDNA in the circulation typically decrease. CtDNA analysis provides a noninvasive method for assessing tumor burden and immune activity.^485–487^ The reduction in ctDNA levels often correlates with effective immune responses, making them useful for monitoring T cell function restoration.

In summary, the restoration of T cell function can be evaluated through a combination of markers, including the CD8^+^ T cell count, activation markers, proliferation indicators, immune cell subsets, and immunosuppressive molecules. These markers provide a comprehensive picture of immune recovery and can guide the effectiveness of immunotherapies, helping clinicians assess T cell reconstitution and optimize treatment strategies.

## Future research directions

### GeNe Editing And Therapeutic Approaches for T cell exhaustion

The emergence of CRISPR–Cas9 technology has radically reshaped genetic engineering, providing unprecedented tools for manipulating genes with precision and efficiency. A promising application of this groundbreaking technology lies in the realm of immunotherapy, particularly in addressing the phenomenon of T cell exhaustion.^488^ TCR modification represents the most straightforward use of CRISPR-Cas9 in immunotherapy (Fig. 4). TCRs are crucial for the recognition of antigens presented by MHC molecules on target cells. In cases where TCRs are ineffective owing to tumor antigen loss or downregulation, CRISPR is used to engineer T cells with enhanced specificity and affinity for tumor-associated antigens.^489^ Moreover, the CRISPR-Cas9 tool enables the knockout of known genes involved in T cell exhaustion, such as *pdcd1* and *ctla4.*^490–492^ CAR and TCR T cells, engineered with disrupted checkpoint molecules, serve as powerful effector cells for combating infectious diseases and cancers.

**Fig. 7:**
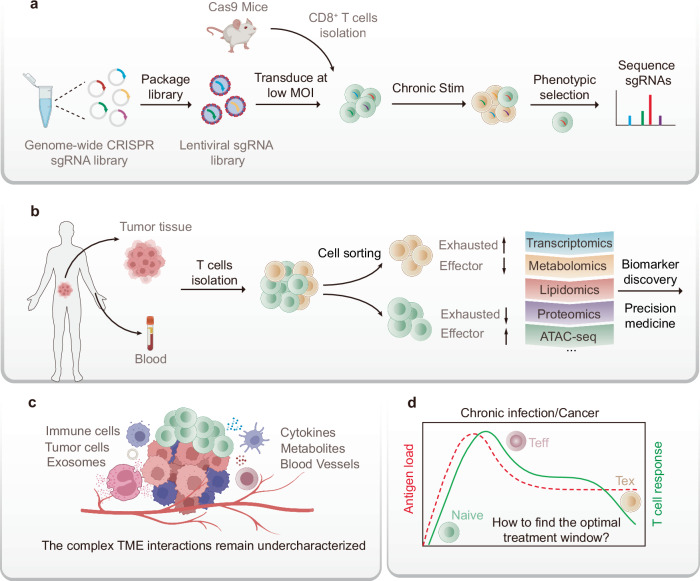
Future research directions in T Cell exhaustion. **a** CRISPR screening can be employed to identify novel genes implicated in T cell exhaustion, offering the potential to discover new molecular targets for therapeutic intervention. **b** The collection of patient-derived T cell samples for comprehensive multi-omic analyses (including transcriptomics, proteomics, and metabolomics) could facilitate the identification of novel biomarkers related to T cell dysfunction and enable the formulation of more customized treatment plans. **c** The tumor microenvironment is crucial for modulating T cell behavior. However, the intricate interactions between T cells and various components of the TME remain insufficiently characterized. Further investigations into these interactions are essential for identifying new therapeutic targets. **d** Longitudinal monitoring of T cell responses in chronic infections and cancer is crucial for understanding the factors driving T cell exhaustion. This approach provides significant insights into the temporal dynamics of T cell dysfunction, helping to identify critical windows for therapeutic intervention

Additionally, CRISPR-Cas9 shows promising potential in the screening of unknown regulatory proteins that contribute to T cell exhaustion, facilitating the identification of therapeutic targets^493,494^ (Fig. 7a). CRISPR screening can be performed in a high-throughput manner, allowing the systematic knockout of genes across the genome to identify those that are critical for specific cellular functions, such as T cell exhaustion. The first step in conducting CRISPR screening is to design a guide RNA (gRNA) library targeting a comprehensive set of genes, and a well-constructed library includes multiple gRNAs per gene to ensure robust targeting and validation. Primary T cells or T cell lines can be transduced with the CRISPR library via lentiviral vectors. This process introduces gRNAs and the Cas9 protein into T cells, resulting in the knockout of targeted genes. Ensuring high transduction efficiency is crucial to achieve sufficient representation of all gRNAs in the population. Following transduction, T cells are subjected to conditions that induce exhaustion. This may involve prolonged stimulation with antigen-presenting cells or exposure to chronic antigen environments, mimicking conditions in cancers or during chronic infections. After the exhaustion-inducing period, T cells are assessed for various functional outcomes. Common assays include measuring cytokine production (e.g., IL-2 and IFN-γ), proliferative capacity, and the expression levels of exhaustion markers (e.g., PD-1 and CTLA-4). The cells are sorted on the basis of these criteria via flow cytometry, allowing for the identification of knockout populations that exhibit restored function. The final step involves analyzing the results to determine which gene knockouts led to enhanced T cell functionality. Bioinformatics tools can be used to correlate gRNA representation with functional readouts, enabling the identification of critical genes connected to T cell exhaustion.

CRISPR-Cas9 technology offers a transformative approach for improving existing immunotherapies through the modification of TCRs and the knockout of known exhaustion-related genes. Moreover, its ability to identify novel regulatory proteins related to T cell dysfunction has facilitated the discovery of new therapeutic targets. With research progress, the application of CRISPR-Cas9 in treating exhausted T cells holds significant promise for advancing immunotherapy, potentially leading to more effective treatments for cancer and chronic infections. By leveraging the strength of this innovative technology, novel personalized and targeted immunotherapeutic strategies can be established, enhancing patient outcomes and revolutionizing cancer treatment.

### Heterogeneity of T cell exhaustion and biomarker discovery

T cell exhaustion is a heterogeneous process that varies across different T cell subsets, tumor types, and individual patients. For example, exhausted CD8^+^ T cells may display distinct molecular and functional characteristics compared with exhausted CD4^+^ T cells. Furthermore, the degree of exhaustion differs substantially even among patients with the same cancer type. Understanding this variability is crucial for developing personalized therapeutic strategies that consider the unique immune profiles of individual patients. To address the heterogeneity of T cell exhaustion, future studies could compare exhaustion signatures across diverse T cell subsets and patient populations (Fig. 7b). Advances in single-cell RNA sequencing and other cutting-edge technologies will aid in uncovering these variations, enabling the identification of specific exhaustion markers that can guide personalized treatment approaches.

Similarly, the discovery and clinical application of novel biomarkers for T cell exhaustion are essential for improving treatment outcomes in patients with cancer and chronic infections. Biomarkers aid in assessing the T cell depletion status, predicting patient responses to therapies, and guiding treatment decisions. Future studies should emphasize the discovery and verification of biomarkers that indicate T cell functionality and predict treatment response. By refining the understanding of these biomarkers, clinicians can better personalize treatment strategies, leading to improved patient outcomes in cancer and chronic infection treatments. These biomarkers could include specific gene expression profiles, protein markers, or metabolic signatures associated with T cell exhaustion.

### TME interactions

The TME profoundly impacts T cell behavior; however, the precise and complex nature of the interactions between T cells and the various cellular and molecular components of the TME remains incompletely understood (Fig. 7c). The TME is a highly dynamic and immunosuppressive environment in which factors such as cytokine gradients, metabolic byproducts, and the presence of immunosuppressive cells collectively contribute to the dysfunction and exhaustion of T cells. Moreover, exploring the cellular and molecular changes within the TME in situ is a significant challenge, primarily due to the complexity and heterogeneity of the TME. Tumors include diverse cell types, including endothelial, stromal, cancer, and immune cells, each of which interact in a dynamic and usually heterogeneous manner. This complexity complicates the capture and analysis of the precise molecular and cellular changes in specific areas of the tumor. Current techniques, such as bulk tissue or single-cell RNA sequencing, provide valuable insights; however, they require tissue dissociation, which results in a loss of spatial context and limits the ability to study the interactions between cells in their native environment. Addressing these challenges requires novel technologies that enable high-resolution, spatially resolved analysis of the TME at the single-cell level and overcome the challenges in visualizing deep tissue structures or capturing transient molecular events. A more comprehensive understanding of TME dynamics could reveal how the TME contributes to T cell exhaustion and help identify novel therapeutic targets, such as cytokine modulators, metabolic inhibitors, or agents that disrupt the function of immunosuppressive cells, offering potential strategies to reinstate T cell function and optimize cancer immunotherapy outcomes.

### Dynamics of T cell exhaustion

Most studies on T cell exhaustion have focused on examining this phenomenon at a single time point, usually overlooking its dynamic nature. T cell exhaustion, particularly in the context of chronic infection or cancer progression, is not a static process. This gradual, time-dependent change is influenced by various factors, including the antigen load, the persistence of inflammatory signals, and alterations in the immune microenvironment. The antigen load, for example, fluctuates over time, with persistent or repeated exposure to a specific antigen causing T cells to progressively diminish their functional capabilities, including cytokine production and cytotoxic activity. Similarly, the immune landscape, which includes factors such as cytokine profiles, the existence of immunosuppressive cells, and metabolic changes, evolves in response to cancer or infection. These shifts in the TME or site of infection may have profound effects on the trajectory of T cell exhaustion. Longitudinal studies that track T cell responses at multiple time points during infection or cancer progression are critical for understanding how exhaustion develops and evolves. These studies provide valuable insights into the molecular and cellular processes driving T cell dysfunction, such as changes in signaling pathways, epigenetic modifications, and the accumulation of inhibitory receptors such as PD-1 and TIM-3. Moreover, such studies can identify critical windows where intervention may be most effective, enabling the development of therapeutic strategies aimed at reversing or preventing T cell exhaustion (Fig. 7d). Understanding the temporal dynamics of T cell exhaustion will allow for the development of more precise and effective approaches to rejuvenating T cells and restoring their antitumor or antipathogen functions, improving immunotherapy outcomes.

## Conclusions

This article reviews the mechanisms of T cell exhaustion, progress in emerging therapies, persisting challenges, and potential avenues for future research. Reversing T cell exhaustion represents a significant frontier in immunotherapy, with promising advancements and significant challenges. The increasing knowledge regarding the mechanisms driving T cell exhaustion has paved the way for innovative therapeutic approaches. Emerging therapies, such as ICIs, cytokine treatments, and metabolic reprogramming strategies, have shown great potential in rejuvenating exhausted T cells and restoring their function in chronic infections and cancer.

Addressing the knowledge gaps in T cell exhaustion research is vital for advancing immunotherapy. Future research could focus on personalized approaches to therapy, identify reliable biomarkers for therapy response prediction, and explore combination strategies to maximize therapeutic effectiveness. The heterogeneity of immune responses among individuals necessitates a personalized approach to therapy, enhancing the effectiveness of immunotherapy and improving patient outcomes. Exploring the complexities of T cell exhaustion should involve considering how individual differences in immune responses may influence susceptibility to exhaustion. With research progress, the integration of personalized strategies could be crucial in reversing T cell exhaustion and achieving durable responses in cancer and chronic infections. Genetic, epigenetic, and environmental factors shape T cell behavior, making it critical to identify biomarkers that predict exhaustion and responsiveness to treatment. Furthermore, combination therapies that address multiple aspects of T cell exhaustion may significantly improve outcomes for patients with chronic infections and cancer.

In conclusion, the exploration of T cell depletion will shape the future of immunotherapy, and basic scientific research can provide guidance and recommendations for clinical treatment, offering patients more effective therapeutic choices in combating some of the most challenging human diseases, particularly in the fields of cancer and infectious and autoimmune diseases.
